# Human milk microbiome: associations with maternal diet and infant growth

**DOI:** 10.3389/fnut.2024.1341777

**Published:** 2024-03-11

**Authors:** Tamara T. Ajeeb, Emmanuel Gonzalez, Noel W. Solomons, Marieke Vossenaar, Kristine G. Koski

**Affiliations:** ^1^School of Human Nutrition, McGill University, Montreal, QC, Canada; ^2^Department of Clinical Nutrition, College of Applied Medical Sciences, Umm Al-Qura University, Makkah, Saudi Arabia; ^3^Canadian Centre for Computational Genomics, McGill Genome Centre, Montreal, QC, Canada; ^4^Department of Human Genetics, McGill University, Montreal, QC, Canada; ^5^Gerald Bronfman Department of Oncology, McGill University, Montreal, QC, Canada; ^6^Center for Studies of Sensory Impairment, Aging and Metabolism (CeSSIAM), Guatemala City, Guatemala

**Keywords:** human breast milk microbiome, breastfeeding, maternal diet, infant growth z-scores, 16S rRNA gene, metagenomics 16S, lactation stage, Guatemala

## Abstract

**Introduction:**

Ingestion of human milk (HM) is identified as a significant factor associated with early infant gut microbial colonization, which has been associated with infant health and development. Maternal diet has been associated with the HM microbiome (HMM). However, a few studies have explored the associations among maternal diet, HMM, and infant growth during the first 6 months of lactation.

**Methods:**

For this cross-sectional study, *Mam*-Mayan mother-infant dyads (*n* = 64) were recruited from 8 rural communities in the Western Highlands of Guatemala at two stages of lactation: early (6–46 days postpartum, *n* = 29) or late (109–184 days postpartum, *n* = 35). Recruited mothers had vaginally delivered singleton births, had no subclinical mastitis or antibiotic treatments, and breastfed their infants. Data collected at both stages of lactation included two 24-h recalls, milk samples, and infant growth status indicators: head-circumference-for-age-z-score (HCAZ), length-for-age-z-score (LAZ), and weight-for-age-z-score (WAZ). Infants were divided into subgroups: normal weight (WAZ ≥ −1SD) and mildly underweight (WAZ < −1SD), non-stunted (LAZ ≥ −1.5SD) and mildly stunted (LAZ < −1.5SD), and normal head-circumference (HCAZ ≥ −1SD) and smaller head-circumference (HCAZ < −1SD). HMM was identified using 16S rRNA gene sequencing; amplicon analysis was performed with the high-resolution ANCHOR pipeline, and DESeq2 identified the differentially abundant (DA) HMM at the species-level between infant growth groups (FDR < 0.05) in both early and late lactation.

**Results:**

Using both cluster and univariate analyses, we identified (a) positive correlations between infant growth clusters and maternal dietary clusters, (b) both positive and negative associations among maternal macronutrient and micronutrient intakes with the HMM at the species level and (c) distinct correlations between HMM DA taxa with maternal nutrient intakes and infant z-scores that differed between breast-fed infants experiencing growth faltering and normal growth in early and late lactation.

**Conclusion:**

Collectively, these findings provide important evidence of the potential influence of maternal diet on the early-life growth of breastfed infants via modulation of the HMM.

## Introduction

The human milk microbiota (HMM) is of growing interest in relation to infant microbial colonization and health outcomes (1, 2). Ingestion of human milk (HM) is considered one of the most significant factors associated with the composition and development of the infant gut microbiome (3–8). More recently, the maternal gut microbiome has emerged as an important source of the HMM (9–13), and although maternal gut microbiome has been shown to be highly influenced by maternal diet (14), only a few studies have explored the association of maternal diet with either the gut or the HMM.

Emerging evidence shows that the maternal diet via the HMM may influence infant growth by shaping the infant gut microbiota (14, 15). The HMM contributes to the establishment of an infant intestinal microbial community (16), and there is evidence that this infant intestinal microbial community is associated with infant growth during early life (17–19). There is also evidence that gut microbiota are associated with postnatal growth in animal models (20). These studies have shown that gut microbiota are involved in metabolic pathways that contribute to postnatal growth directly through energy harvesting (21), synthesizing vitamins (22), and affecting growth hormone and somatotropic axis sensitivity (20), and indirectly by regulating the immune system and preparing infants to face environmental challenges to their health (22).

Several studies have explored the association of the maternal diet/nutrients with the HMM, revealing multiple positive and negative associations between maternal micronutrient intakes and the HMM of lactating mothers (23–26). However, associations of HMM with infant growth mediated by maternal diet have not been explored. Maternal diet is known to affect the maternal gut microbiome community (27–32), which is a major source of the HMM (9, 10, 33). This is supported by evidence of the vertical transmission of the maternal milk microbiome and by shared bacterial species between paired maternal fecal and/or milk and infant fecal samples (3–5, 9, 34).

Guatemala is a developing country, with high exclusive breastfeeding rates approaching 77% (35). Moreover 90% of Indigenous mothers continue to breastfeed beyond 6 months for 17–21 months compared with 41.7% in the general population (36). The most recent available UNICEF 2015 Children Statistics for Guatemala reported that approximately 47% of Guatemalan children younger than 5 years of age were stunted (37). Although the Indigenous population of Guatemala complies with the WHO recommendations to exclusively breastfeed during the first 6 months of the infant’s life (38, 39), earlier reports showed that the growth faltering occurs soon after birth in rural Guatemala (40) and is present for 3–6 months among breastfed Guatemalan infants (41). However, the association of the HMM with infant growth has been investigated in one study only (42). To date, no studies have explored the association between maternal diet and HMM in early and late lactation, both of which have been shown to have different HMM community composition (43), or examined potential associations of HMM with infant growth that may be mediated by maternal diet.

Recently, we reported differences in the HMM between infants with normal growth and infants with mild growth faltering (44, 45). We identified 30 differentially abundant (DA) taxa between the LAZ groups [mildly stunted (LAZ < −1.5SD) versus the non-stunted (LAZ ≥ −1.5SD)], 23 DA taxa between the WAZ groups [normal weight (WAZ ≥ −1SD) versus the mildly underweight (WAZ < −1SD)], and 26 DA taxa were identified between HCAZ groups [normal HC (HCAZ ≥ −1 SD) versus the smaller HC (HCAZ < −1 SD)] (44, 45). Our present study aimed to assess the association between maternal nutrient intakes and the DA HMM taxa identified at early and late lactation between infants with normal growth compared with infants with mild growth faltering among exclusively breastfed mother–infant dyads.

## Materials and methods

### Study setting, recruitment, and ethics

This cross-sectional study was conducted in eight rural *Mam*-Mayan communities of the Western Highland departments of Quetzaltenango between June 2012 and January 2013 (46). The *Mam*-Mayan community constitutes the fourth-largest Mayan population in Guatemala (47). The study began as collaboration between McGill University and the Center for Studies of Sensory Impairment, Aging, and Metabolism (CeSSIAM), a research organization based in Guatemala. Ethical approvals were obtained from ethics boards at McGill University and CeSSIAM. Further approvals were obtained from community leaders and the local authorities at the Ministry of Health in Guatemala. Community health workers used a participatory action research framework (48) to recruit lactating mothers through home visits, loudspeaker announcements, and word-of-mouth invitations (46). Recruited mothers provided fully informed written consent (thumbprint if unable to sign) if they wished to participate, and all mothers were informed of their rights to withdraw at any time from the study.

### Study design

This cross-sectional study included healthy mother–infant dyads from two stages of lactation: “early” (6–46 days postpartum) or “late” (109–184 days postpartum). Inclusion criteria were healthy mother–infant dyads aged 6–46 days and 109–184 days postpartum, mothers who delivered vaginally, breastfed their infants exclusively or predominantly (used *agüitas*, a ritual fluid) for 6 months, and provided two 24-h dietary recalls at each stage of lactation. Exclusion criteria were non-singleton births, mother–infant dyads younger than 4 days, due to the possibility of still feeding colostrum, or did not have sufficient milk volume for the analysis, mothers treated with antibiotics, and mothers with sub-clinical mastitis (Na: K > 0.6) due to the possible effect of sub-clinical inflammation on the milk microbiome community and infant growth (49). Data included infant growth parameters, maternal anthropometries, maternal nutrient intakes (energy, macronutrients, and vitamins), and the DA species associated with infant z-scores.

### Infant anthropometry

Infant age and infant anthropometric measurements (height, weight, and head circumference) were recorded by two trained Guatemalan nutritionists according to the standardized procedures. The detailed methodology was previously published (46). In brief, infant age was either calculated from the date of birth recorded on the maternal health card or was obtained from the mother in the absence of the health card. Infant anthropometric measurements were measured thrice, and the final value was the calculated mean of the three measurements. Infant recumbent supine length (cm) was measured thrice using an infant meter, a mobile baby measuring mat (SECA 210), and the calculated mean was rounded to the nearest 0.5 cm. Infant weight (kg) was measured using a digital infant scale (SECA 354) and rounded to the nearest 100 g. Finally, infant head-circumference (cm) was measured thrice using a head-circumference baby band (SECA 212). All infant anthropometric measures were completed on the same day of the milk sample collection.

Infant growth status indicators were calculated using the World Health Organization Anthro software (3.1) (50) for length-for-age z-score (LAZ), weight-for-age z-score (WAZ), and head circumference-for-age z-score (HCAZ) at early and late lactation. In brief, infants were divided into comparison groups: ‘non-stunted’ [LAZ ≥ −1.5SD (early: *n* = 11; late: *n* = 16)] versus ‘mildly stunted’ [LAZ < −1.5SD (early: *n* = 18; late: *n* = 19)], ‘normal weight’ [WAZ ≥ −1SD (early: *n* = 20 late: *n* = 20)] versus ‘mildly underweight’ [WAZ < −1SD (early: *n* = 9; late: *n* = 15)], and ‘normal head-circumference’ [HCAZ ≥ −1SD (early: *n* = 19 late: *n* = 16)] versus ‘smaller head-circumference’ [HCAZ < −1SD (early: *n* = 10; late: *n* = 18)]. The threshold of 1SD (i.e., more than 1SD below the WHO standard median) is used to define mild growth faltering. However, we used LAZ < −1.5SD to define mild stunting instead of LAZ < −1SD, to avoid over-estimation of both stunting prevalence and the association between the HMM and stunting, as stunting at birth and during the first month of life (4–33 days, median: 19 days) has been reported among Guatemalan infants (51).

### Maternal diet records

Staff nutritionists conducted 2 comprehensive quantitative non-consecutive days of 24-h recalls in Spanish or Mam in both early and late lactation, as previously described (46). All foods and beverages were recorded and included in the analysis, and mothers were not taking food or vitamin supplements. National food composition tables for Central America from the Institute of Nutrition of Central America and Panama (INCAP) (52) were used to establish maternal intakes for energy, percent of energy from carbohydrates, protein, and total fat, macronutrient intakes for saturated fatty acids, monounsaturated fatty acids, polyunsaturated fatty acids, cholesterol, fiber, sugar, and micronutrient intakes for vitamins including vitamin C, thiamin, riboflavin, niacin, pantothenic acid, pyridoxine, folate, cobalamin, choline, vitamin A, retinol, alpha-carotene, beta-carotene, beta-cryptoxanthin, lutein + zeaxanthin, vitamin E, Vitamin D, and vitamin K. To estimate usual maternal intakes of energy, macronutrient intakes, and vitamins, average intakes of the 2 non-consecutive days of 24-h recalls for each nutrient were calculated at each stage of lactation. Maternal nutrient intakes were transformed using the nutrient density method where maternal macronutrient intakes were calculated as the proportion of total energy intake from carbohydrates, protein, and fat (e.g., % kcal from total fat), and micronutrient intakes were calculated in typical units per 1,000 kcal. This nutrient density method has been used among women in low-income, urban settings across three countries and has high probability of identifying inadequate intakes for several micronutrients (53). Due to a previously published report identifying true low intakes among our study population related to poverty (46), underreporting was not applied, and no dietary record was removed. Maternal dietary intakes were also compared with the Acceptable Macronutrient Distribution Range (AMDR).

### Human milk sample collection

Milk samples were collected on the same day as the second 24-h recall to increase the accuracy of assessing the correlation between maternal diet and the human milk microbiome (HMM). To minimize the possibility of exchanging microbes, mothers were recruited from eight distinct remote communities. Mothers in the *Mam*-Mayan communities are known to comply with WHO recommendations to exclusively or predominantly breastfeed for the first 6 months of the infant’s life (38, 39). Milk samples were collected from all mothers unilaterally from the breast that was not last used to feed the infant by full manual expression in a 3-h time window between 9 am and 12 pm (38). All milk samples were collected following an aseptic technique by a trained midwife, who used hand sanitizer before and after collection. The nipple and areola of the breast were cleaned with 70% ethanol prior to milk sample collection. Only manual expression was used for milk collection, without the use of a breast pump to exclude its potential influences on milk microbiome diversity (54). Milk samples were collected in acid-washed, sterile 60-ml plastic vials and stored on ice immediately and subsequently partitioned into four 15 mL vials and stored at −30°C in the field laboratory. Samples were shipped to McGill University, where they were stored at −80C (55), which is an optimum temperature for microbiome preservation prior to DNA extraction (56).

### 16S rRNA gene amplification and sequencing

A DNeasy Blood and Tissue mini kit from QIAGEN was used with 1 mL of milk to extract DNA, according to the manufacturer’s protocol by Génome Québec laboratories. PCR amplification was conducted with the universal eubacteria primers 27F/533R (27F: AGAGTTTGATCCTGGCTCAG, 533R: TTACCGCGGCTGCTGGCAC) of the variable regions V1–V3 consisting of ~526 bp based on the *Escherichia coli* 16S rRNA gene (57–59). These primers have a high coverage of most genera currently considered “core” in human milk, including the genus *Cutibacterium* (60, 61). Sequencing was performed using the Illumina MiSeq platform. Reagent controls were below the detection limit. The amplification conditions have been previously described (43).

Contamination control steps were performed at multiple steps in this analysis. At the milk sample collection stage, a trained midwife followed an aseptic sampling protocol which included cleaning hands with hand sanitizers and using 70% ethanol prior to cleaning the nipple and areola of the breast prior to milk sample collection. At the PCR step, the Genome Quebec Centre followed an aseptic technique.

### Microbial data processing and bioinformatics

Analysis of the amplicon data was performed using the ANCHOR pipeline. ANCHOR is a method designed for improved species-level microbial identification through the utilization of direct paired-end sequences, which substantially improves the sequence resolution of 16 s rRNA amplification data. Furthermore, it uses multiple samples and integrated multiple-reference databases to annotate bacteria with criteria of >99% for identity and coverage to provide high confidence and resolution (62).

In brief, Mothur was used to align DE replicate sequences (63) before high-count ESV selection at a count threshold of 36 across all samples. The repository databases such as NCBI 16S rRNA RefSeq, NCBI non-redundant nucleotide, SILVA, and the Ribosomal Database Project (RDP) were used to annotate ESVs using BLASTn with criteria of >99% for identity and coverage. Priority was given to NCBI 16S rRNA RefSeq when BLASTn, 100% identity, and coverage hits were returned across multiple databases due to the high standard of curation. Amplicons with low counts (<36) were binned to high-count ESVs at a threshold of >98% identity/coverage. Multiple, equally good (highest identity/coverage), annotation was retained and reported as a multiple species (_*MS*). Taxonomy annotation, particularly species calls, should be considered putative even when sharing 100% sequence identity to a single species due to database errors.

Several contamination control steps via sample pre-processing were performed by the Canadian Centre for Computational Genomics (C3G) of McGill University at the bioinformatics stage, including controlling for prevalence and sparsity, ordination analysis, and identifying putative contamination, which is flagged by *Decontam* (*Decontam*, R package) (Supplementary file 1). *Decontam* flags putative contamination. Out of 1,505 ESVs, only one ESV was flagged as potential contamination, although it was not selected by DESeq2 as a differentially abundant species in our samples.

### Statistical analyses

Initially, we assessed if maternal age and anthropometries (weight, height, and BMI) were associated with infant z-scores (WAZ, LAZ, WHZ, BMIZ, and HCAZ) in both stages of lactation. Thereafter, infant anthropometry and maternal nutrient intakes were compared for LAZ, WAZ, and HCAZ between infants with HCAZ ≥ −1 SD, LAZ ≥ −1.5SD, and WAZ ≥ −1SD compared with those infants with smaller head circumferences (HCAZ < −1 SD) and those with mild linear growth deficits (LAZ < −1.5SD) and those classified as mildly underweight (WAZ < −1SD); comparisons used *t*-tests or non-parametric Wilcoxon tests and chi-square for continuous and categorical variables, respectively. To describe population characteristics, anthropometric (Table 1) and dietary (Table 2) data were presented as means ± standard deviations (SD) for continuous variables. A *p-*value <0.05 was considered significant for these analyses.

**Table 1:** Population characteristics of Guatemalan mother-infant dyads.

Characteristic	HCAZ ≥ −1 SD *n* = 34^1^	HCAZ < −1 SD *n* = 30^1^	*p*-value^2^	LAZ ≥ −1.5 SD *n* = 27^1^	LAZ < −1.5 SD *n* = 37^1^	*p*-value^2^	WAZ ≥ −1 SD *n* = 40^1^	WAZ < −1 SD *n* = 24^1^	*p*-value^2^
Lactation stage			0.2			0.5			0.3
Early	18 (53%)	11 (37%)		11 (41%)	18 (49%)		20 (50%)	9 (38%)	
Late	16 (47%)	19 (63%)		16 (59%)	19 (51%)		20 (50%)	15 (62%)	
Maternal characteristics									
Maternal age, years	24 (6.4)	23.4 (5.38)	0.8	24.1 (6)	23.4 (5.9)	0.6	23.3 (5.4)	24.3 (6.8)	0.8
Maternal height	147.6 (5.5)	146.6 (4.4)	0.4	148.2 (5.1)	146.3 (4.9)	0.15	148 (4.8)	145.7 (5.7)	**0.038**
Maternal weight	52.4 (8.3)	50.4 (7.1)	0.4	53.3 (7.7)	50.2 (7.7)	0.13	52.2 (7.3)	50.2 (8.4)	0.4
Maternal BMI	24 (3.1)	23.5 (3.6)	0.7	24.2 (3.2)	23.4 (3.4)	0.3	24 (3.3)	23.6 (3.5)	0.8
Infant characteristics									
Infant sex			0.8			0.14			0.2
Female	17 (50%)	16 (53%)		11 (41%)	22 (59%)		18 (45%)	15 (62%)	
Male	17 (50%)	14 (47%)		16 (59%)	15 (41%)		22 (55%)	9 (38%)	
Infant age, days	81 (64)	97 (61)	0.6	93 (60)	85 (65)	0.6	83 (66)	97 (58)	0.4
Infant WAZ	−0.46 (0.7)	−1.17 (0.95)	**0.001**	−0.28 (0.75)	−1.17 (0.79)	**<0.001**	−0.29 (0.56)	−1.63 (0.68)	**<0.001**
Infant LAZ	−1.4 (1.03)	−2.27 (1.17)	**0.008**	−0.8 (0.68)	−2.54 (0.87)	**<0.001**	−1.39 (1)	−2.5 (1.12)	**<0.001**
Infant WHZ	0.87 (0.9)	0.71 (1.32)	>0.9	0.43 (0.97)	1.06 (1.14)	**0.013**	1 (1.02)	0.47 (1.2)	0.15
Infant HCAZ	−0.09 (0.57)	−1.9 (1.03)	**<0.001**	−0.53 (1.2)	−1.2 (1.17)	**0.019**	−0.50 (1)	−1.62 (1.23)	**<0.001**
Infant BMIZ	0.46 (0.78)	0.18 (1.08)	0.4	0.22 (0.96)	0.41 (0.93)	0.5	0.7 (0.76)	−0.27 (0.91)	**<0.001**
Mild Underweight	7 (21%)	17 (57%)	**0.003**	5 (19%)	19 (51%)	**0.007**	-	-	-
Mild Stunting	15 (44%)	22 (73%)	**0.018**	-	-	**-**	18 (45%)	19 (79%)	**0.007**
Smaller head circumference	-	-	-	8 (30%)	22 (59%)	**0.018**	13 (32%)	17 (71%)	**0.003**

^1^n/N (%); Mean (SD). ^2^Pearson’s Chi-squared tests; Wilcoxon rank sum test; Wilcoxon rank sum exact test. HCAZ: head-circumference-for-age-z-score, LAZ: length-for-age-z-score, WAZ: weight-for-age-z-score, BMI: body mass index, WHZ: weight-for-height-z-score, BMIZ: body mass index-z-score. The bold values are statistically significant *p* < 0.05.

**Table 2:** Maternal nutrient intakes by infant growth z-scores.

Nutrient intakes	HCAZ ≥ −1 SD *n* = 34^1^	HCAZ < −1 SD *n* = 30^1^	*p*-value^2^	LAZ ≥ −1.5 SD *n* = 27^1^	LAZ < −1.5 SD *n* = 37^1^	*p*-value^2^	WAZ ≥ −1 SD *n* = 40^1^	WAZ < −1 SD *n* = 24^1^	*p*-value^2^
Energy kcal	1,508 (243)	1,395 (275)	0.2	1,435 (329)	1,470 (205)	0.7	1,517 (247)	1,353 (260)	**0.018**
Protein g	46 (11)	43 (14)	0.4	44 (14)	45 (12)	0.6	46 (12)	43 (14)	0.4
Carbohydrates g	295 (55)	278 (54)	0.4	281 (73)	292 (36)	0.5	298 (55)	269 (51)	**0.049**
Fat g	24 (7)	20 (6)	0.081	23 (8)	22 (6)	0.4	24 (7)	20 (6)	**0.016**
Saturated fat g	5 (3)	4 (2)	0.3	5 (3)	4 (2)	0.4	5 (3)	4 (2)	0.069
Monounsaturated fat g	7 (3)	6 (2)	**0.049**	7 (3)	7 (2)	0.5	7 (2.6)	6 (2)	**0.016**
Polyunsaturated fat g	9 (2)	7 (2)	0.061	8 (2)	8 (2)	0.7	8 (2)	7 (2)	**0.026**
Cholesterol mg	97 (95)	85 (87)	0.6	94 (105)	90 (81)	0.6	105 (95)	70 (81)	0.2
Fibers g	30 (7)	28 (6)	0.9	28 (8)	30 (5)	0.3	30 (6)	28 (7)	0.4
Sugar g	77 (30)	64 (25)	0.2	70 (33)	71 (24)	0.4	75 (31)	64 (22)	0.2
Vitamin C mg	57 (35)	45 (27)	0.2	48 (31)	54 (33)	0.6	49 (30)	55 (36)	0.7
Thiamin mg	0.89 (0.24)	0.82 (0.2)	0.4	0.83 (0.26)	0.88 (0.2)	0.3	0.87 (0.22)	0.84 (0.23)	0.7
Riboflavin mg	0.71 (0.23)	0.66 (0.22)	0.6	0.68 (0.25)	0.69 (0.21)	0.7	0.73 (0.22)	0.62 (0.21)	0.058
Niacin mg	15.5 (3.7)	15.5 (6.1)	0.5	15.1 (6.1)	15.8 (4)	0.6	15.5 (4)	15.5 (6.3)	0.4
Pantothenic acid mg	2.6 (0.85)	2.5 (0.74)	0.5	2.5 (0.92)	2.63 (0.71)	0.8	2.69 (0.76)	2.4 (0.85)	0.2
Vitamin B6 mg	1.2 (0.32)	1.1 (0.41)	0.3	1.1 (0.36)	1.2 (0.38)	0.9	1.2 (0.37)	1.1 (0.37)	0.5
Folate DFE μg	528 (138)	490 (116)	0.4	503 (158)	516 (104)	0.6	527 (127)	483 (129)	0.4
Choline mg	183 (85)	177 (87)	0.9	181 (97)	179 (78)	>0.9	189 (81)	164 (91)	0.2
Vitamin B12 μg	0.74 (0.62)	0.71 (0.60)	>0.9	0.83 (0.72)	0.65 (0.50)	0.4	0.77 (0.60)	0.66 (0.63)	0.4
Vitamin A RAE	983 (326)	841 (410)	0.084	865 (377)	954 (368)	0.4	962 (371)	840 (366)	0.2
Retinol μg	567 (266)	433 (252)	0.055	488 (293)	515 (248)	0.3	548 (272)	430 (243)	0.10
Alpha-carotene μg	792 (771)	704 (964)	0.2	653 (743)	821 (941)	>0.9	742 (828)	765 (932)	>0.9
Beta-carotene μg	2,675 (2,231)	2,632 (2,563)	0.8	2,349 (2,164)	2,878 (2,520)	0.5	2,592 (2,300)	2,760 (2,536)	0.9
Beta-cryptoxanthin μg	171 (541)	203 (601)	0.6	70 (163)	271 (723)	0.2	245 (690)	88 (226)	0.7
Lycopene μg	338 (560)	427 (1,084)	0.3	247 (383)	477 (1,053)	0.9	286 (501)	537 (1,212)	0.4
Lutein + zeaxanthin μg	1,663 (1,828)	1,979 (2,054)	0.2	1,821 (1,998)	1,804 (1,903)	0.6	1,695 (1,657)	2,006 (2,337)	0.8
Vitamin E mg	3.4 (1.8)	2.3 (1)	**0.003**	2.8 (1.4)	2.9 (1.7)	0.9	3.2 (1.7)	2.34 (1)	**0.029**
Vitamin D μg	0.6 (0.9)	0.5 (0.7)	0.8	0.6 (0.9)	0.5 (0.7)	0.6	0.7 (0.9)	0.28 (0.5)	0.060
Vitamin K μg	86 (97)	84 (90)	0.7	92 (105)	80 (84)	0.8	81 (89)	92 (101)	0.6

^1^n/N (%); Mean (SD). ^2^Pearson’s Chi-squared tests; Wilcoxon rank sum test; Wilcoxon rank sum exact test. HCAZ: head-circumference-for-age-z-score, LAZ: length-for-age-z-score, WAZ: weight-for-age-z-score, BMI: body mass index, WHZ: weight-for-height-z-score, BMIZ: body mass index-z-score. The bold values are statistically significant *p* < 0.05.

Correlation analyses were performed to assess the relationships among maternal anthropometry and infant growth parameters, the dependency among maternal nutrient intakes, and among maternal anthropometry and infant growth parameters with maternal dietary intake per nutrient in early and late lactation (FDR < 0.05).

Spearman’s rank-order correlation coefficients were calculated using normalized ESV abundance and the infant growth parameters from mildly stunted (LAZ < −1.5SD), non-stunted (LAZ ≥ −1.5SD), mildly underweight (WAZ < −1SD), normal weight (WAZ ≥ −1SD), normal head-circumference (HCAZ ≥ −1SD), and smaller head-circumference (HCAZ < −1SD) groups with the average of two 24-h recalls of maternal nutrient intakes in early and late lactation. Correlation coefficients were represented by heatmaps using the *corrplot* v0.92 R package. To control for multiple comparisons, a false discovery rate (FDR) was applied to *p*-values (FDR < 0.1).

Mantel correlations were obtained by performing Mantel test between distance matrices of clustered phenotypic values (maternal nutrients, infant growth parameters) and ESVs. Clusters were selected manually from heatmaps by applying a hierarchical clustering order (hclust option in *corrplot*) on each group correlation. Distance matrices were calculated by using Euclidean distance measure for phenotypic value and Jaccard distance (*vegdist* function in the vegan R package) of rlog-transformed ESV abundance.

## Results

### Population characteristics and maternal nutrient intakes of Guatemalan mother–infant dyads

#### Overall assessment of quality of maternal diet

Maternal macronutrient intakes of the majority of the lactating mothers were imbalanced and fell outside the Acceptable Macronutrient Distribution Range (AMDR) (64). The AMDR for adults for carbohydrate ranges from 55 to 70%, for protein from 7 to 20%, and for fat from 15 to 25% of the daily energy intake. In our study, Guatemalan mothers’ carbohydrate intakes constituted more than 70% of the daily energy intakes for 95.3% of mothers and most (91.7%) had intakes of fat below its AMDR. For protein, only 14.1% of mothers consumed proteins ≥15% of total energy intake. Mothers also had inadequate intakes of multiple vitamins when compared with two-thirds of the Recommended Dietary Allowances (RDA) or the national RDA known as *Recomendaciones Dietéticas Diarias* (RDD) (65, 66). The prevalence of inadequate intakes based on INCAP included thiamin (50%), riboflavin (94%), pyridoxine (70%), folate (97%), and cobalamin (90%). The mean energy intake among the Guatemalan mothers in our study was 1,455 calories/day which was uniformly lower than the estimated mean energy requirements of 2,065 calories per day based on estimated resting energy expenditure using the Dietary Reference Intake (DRI) equation of the Institute of Medicine (67, 68) and a moderate physical activity factor of 1.7 (69).

#### Association of anthropometry with maternal diet and nutrient intakes

Maternal–infant dyad anthropometric characteristics are shown in Table 1. Differences in maternal dietary intakes by infant z-scores: HCAZ ≥ −1SD versus smaller head-circumference (HCAZ < −1SD), LAZ ≥ −1.5SD versus mildly stunted (LAZ < −1.5SD), and WAZ ≥ −1SD versus mildly underweight (WAZ < −1SD) are shown in Table 2.

Maternal anthropometries were not associated with infant z-scores in either early (*p* = 0.6) or late (*p =* 0.8) stages of lactation. On the other hand, comparisons showed that infants with HCAZ < −1 SD had a higher prevalence of mild stunting (*p* = 0.018) and mild underweight (*p* = 0.003) compared with infants with HCAZ ≥ −1 SD. These mothers also consumed less MUFA (*p* = 0.049), vitamin E (*p* = 0.003), and marginally less retinol (*p* = 0.055) compared with the mothers of infants with HCAZ ≥ −1 SD. Mildly stunted (LAZ < −1.5SD) infants had a higher prevalence of mild underweight (*p* = 0.007) and had smaller head-circumferences (*p* = 0.018) compared with infants with LAZ ≥ −1.5SD. However, maternal intakes of the studied nutrients did not differ between infant LAZ groups. For the comparison of infants classified as having WAZ < −1SD, there was a higher prevalence of mild stunting (*p* = 0.007) and smaller head-circumferences (*p* = 0.003) compared with infants with WAZ ≥ −1SD. For these infants, maternal intakes of energy (*p* = 0.018), carbohydrates (*p* = 0.049), fat (*p* = 0.016), and dietary fat components, including MUFA (*p* = 0.016), PUFA (*p* = 0.026), and vitamin E (*p* = 0.029), were lower for mothers of mildly underweight (WAZ < −1SD) infants compared with maternal intakes for infants with WAZ ≥ −1SD (Tables 1, 2).

### Cluster analyses in early and late lactation

#### Maternal and infant anthropometric correlations

**Figure 1:**
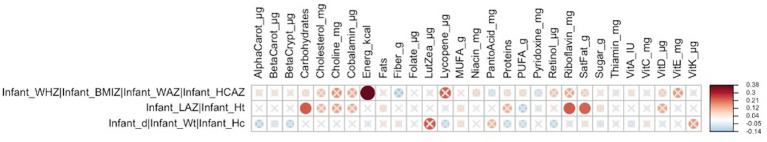
Heatmap of Mantel tests between maternal nutrient intakes (early lactation; Euclidean distance metric) and maternal and infant anthropometry clusters (based on Bray-Curtis distance metric). Red circles represent positive correlations and blue circles represent negative correlations. The intensity of the colors represents the degree of association. The solid circles represent significant correlations (FDR > 0.1). Cluster 1: infant weight-for-height-z-score (WHZ), infant BMI-for-age-z-score (BMIZ), infant weight-for-age-z-score (WAZ), and infant head-circumference-for-age-z-score (HCAZ) was significantly correlated with maternal energy intakes (FDR = 0.029; *r* = 0.38), and Cluster 2: infant-length-for-age-z-score (LAZ), and infant height was significantly correlated with maternal intakes of carbohydrates (FDR = 0.093; *r* = 0.23), riboflavin (FDR = 0.093; *r* = 0.23), and saturated fat (FDR = 0.093; *r* = 0.22).

In early lactation, the cluster of infant z-scores (WHZ, BMIZ, WAZ, and HCAZ) (Supplementary Figure S1) was correlated with maternal nutrient intakes including energy intake (FDR = 0.029; *r* = 0.4), and the infant linear growth cluster (LAZ and length/height) was correlated with maternal intakes of carbohydrates (FDR = 0.093; *r* = 0.23), riboflavin (FDR = 0.093; *r* = 0.23), and saturated fat (FDR = 0.093; *r* = 0.22) (Figure 1).

**Figure 2:**
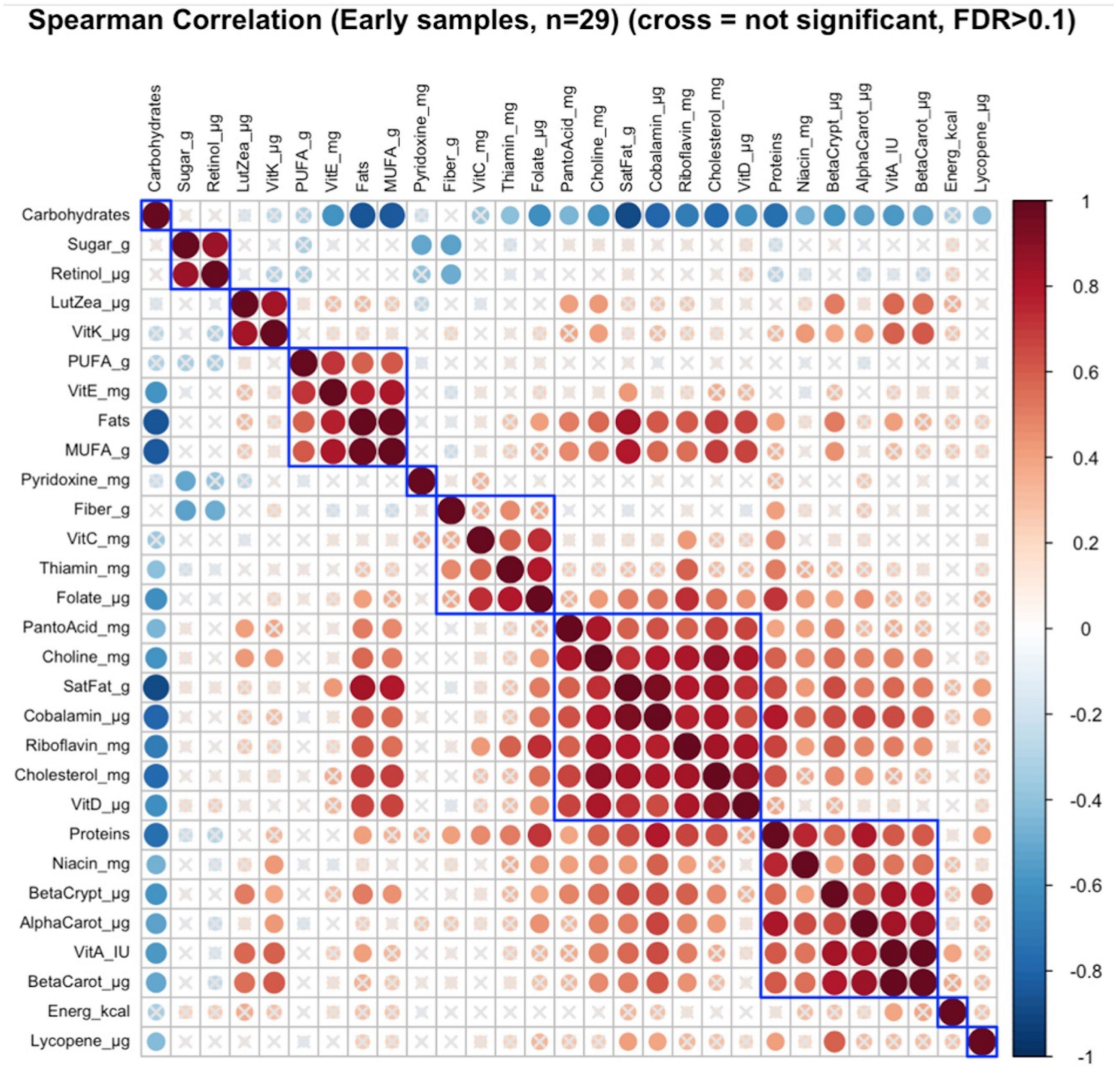
Hierarchical clustering of Spearman’s rank correlation heatmap between maternal nutrient intakes in early lactation. Red circles represent positive correlations and blue circles represent negative correlations. The blue boxes represent inferred clusters. Cluster 1: maternal intakes of sugar and retinol, Cluster 2: maternal intakes of lutein + zeaxanthin and vitamin K, Cluster 3: maternal intakes of polyunsaturated fatty acids (PUFA), vitamin E, fats, and monounsaturated fatty acids (MUFA), Cluster 4: maternal intakes of fiber, vitamin C, thiamin, and folate, Cluster 5: maternal intakes of pantothenic acid, choline, saturated fatty acids, cobalamin, riboflavin, cholesterol, and vitamin D, and Cluster 6: maternal intakes of proteins, niacin, beta-cryptoxanthin, alpha-carotene, vitamin A, and beta-carotene.

Of the six distinct nutrient clusters that were identified in early lactation (Figure 2), there was a significant correlation between maternal nutrient intakes of lutein + zeaxanthin and vitamin K as a cluster with the infant growth parameters (infant age, infant weight, and infant head-circumference) as a cluster (FDR = 0.08; *r* = 0.23) (Supplementary Figure S2).

**Figure 3:**
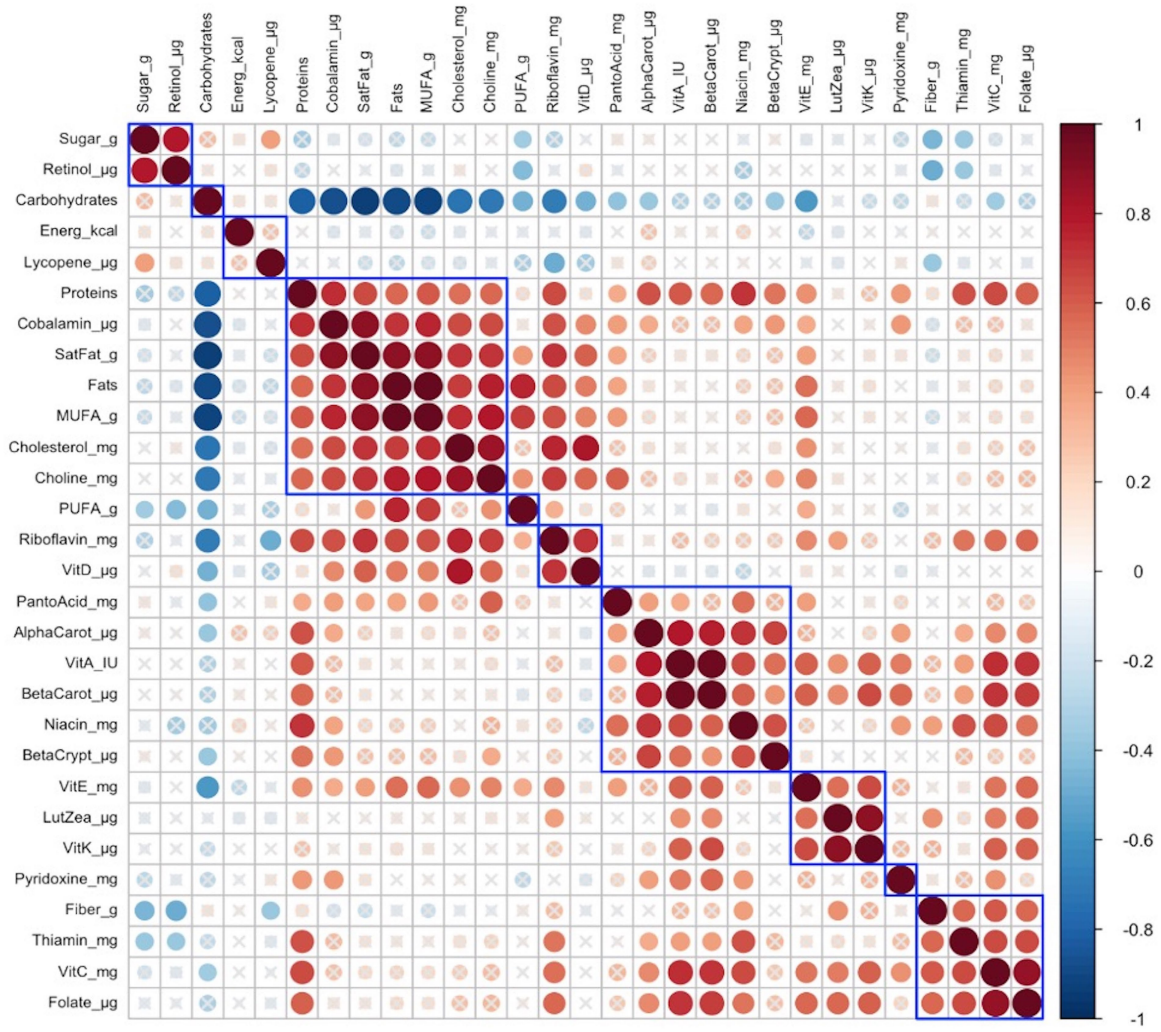
Hierarchical clustering of Spearman’s rank correlation heatmap between maternal nutrient intakes in late lactation. Red circles represent positive correlations and blue circles represent negative correlations. The blue boxes represent inferred clusters. Cluster 1: maternal intakes of retinol, and sugar, Cluster 2: maternal intakes of protein, cobalamin, saturated fat, fats, monounsaturated fatty acids (MUFA), cholesterol, and choline, Cluster 3: maternal intakes of riboflavin and vitamin D, Cluster 4: maternal intakes of pantothenic acid, alpha-carotene, vitamin A, beta-carotene, niacin, and beta-cryptoxanthin, Cluster 5: maternal intakes of vitamin E, lutein + zeaxanthin, and vitamin K, and Cluster 6: maternal intakes of fiber, thiamin, vitamin C, and folate.

In late lactation, no infant anthropometric cluster was correlated with any identified maternal nutrient intake cluster is shown in Figure 3.

#### Identification of nutrient patterns and clusters in early and late lactation

Correlation analyses of individual maternal nutrient intakes and nutrient clusters in early and late lactation are shown in Figure 2 for early lactation and in Figure 3 for late lactation. In both early and late lactation energy intakes were not correlated with intakes of other nutrients, but higher dietary intakes of carbohydrate as a percent of total kcal were inversely correlated with individual energy intakes from protein and total fat, also inversely correlated with gram intakes of saturated fats as MUFA or PUFA and cholesterol and finally with lower intakes of several micronutrients including fat soluble (E, D and/or A, K) and multiple water-soluble vitamins.

Cluster correlation analyses of maternal nutrient intakes in early (Figure 2) lactation and late (Figure 3) lactation represented inferred nutrient clusters with some differences between the clusters in early lactation and late lactation. First, an interesting cluster, possibly unique to the Guatemalan diet, emerged where sugar was clustered with retinol. In Guatemala, sugar is fortified with retinol, which was associated with lower intakes of fiber and pyridoxine in early and lower intakes of fiber and thiamine in late lactation. The second common cluster was of three vitamins—ascorbic acid, thiamine, and folate—with fiber. The third common cluster was lutein + zeaxanthin and vitamin K, which emerged as a cluster in early lactation; in late lactation, vitamin E was also emerged as positively correlated with this cluster. The fourth common cluster was vitamin A, its precursors were alpha-carotene, beta-carotene, and beta-cryptoxanthin with niacin. The last common cluster was choline, saturated fat, cobalamin, and cholesterol.

Despite similarities, differences occurred in the nutrient composition of clusters between early and late lactation. These clusters were associated with protein as a percentage of kcal, fat as a percentage of kcal, gram intakes of MUFA and PUFA, and mg intakes of vitamin D, vitamin E riboflavin, and pantothenic acid. A cluster with fat, MUFA, PUFA, and vitamin E in early lactation emerged, but a related cluster did not appear in late lactation, where fats and MUFA were positively clustered with a larger number of nutrients including higher intakes of protein as a percentage of kcal, saturated fats, cholesterol, choline, cobalamin, and nutrients often associated with intakes of animal source foods. In addition, in early lactation, protein was clustered with some nutrients that were related to plant intake including, beta-cryptoxanthin, alpha-carotene, and beta-carotene, and another two nutrients found in both plant and animal sources, vitamin A, and niacin. The opposite was true for pantothenic acid, which was clustered with animal-related nutrients including, choline, saturated fat, cobalamin, riboflavin, cholesterol, and vitamin D in early lactation, whereas it was clustered with more plant-related nutrients including alpha-carotene, vitamin A, beta-carotene, niacin, and beta-cryptoxanthin in late lactation.

### Correlations of the human milk microbiome with maternal nutrient intakes

#### Human milk microbiome community

ANCHOR was able to identify 503 ESVs and captured 3,551,788 sequence reads across 64 human milk samples. Among the identified 503 ESVs, 256 were annotated at the species level, accounting for 81.2% of reads, 129 were annotated at the genera level, and 9 at the family-level or higher taxa in addition to 109 unidentified taxa that accounted for 6.5% of the total ESVs. These taxa were classified as Unknowns as they could not be identified at >99% similarity in both identity and coverage to any known taxa. There were also 67 ambiguous species that were given the suffix (_*MS*) ‘Multiple Species’. The suffix was used for ESVs when multiple species are equally likely annotated.

#### Human milk microbiome and maternal nutrient intakes

Correlation analyses are illustrated in heatmaps and identified significant associations between maternal dietary intakes and HMM at the species level in early (Supplementary File 2) and late (Supplementary File 3) lactation.

#### Cluster correlations of DA ESVs and maternal nutrient intakes

**Figure 4:**
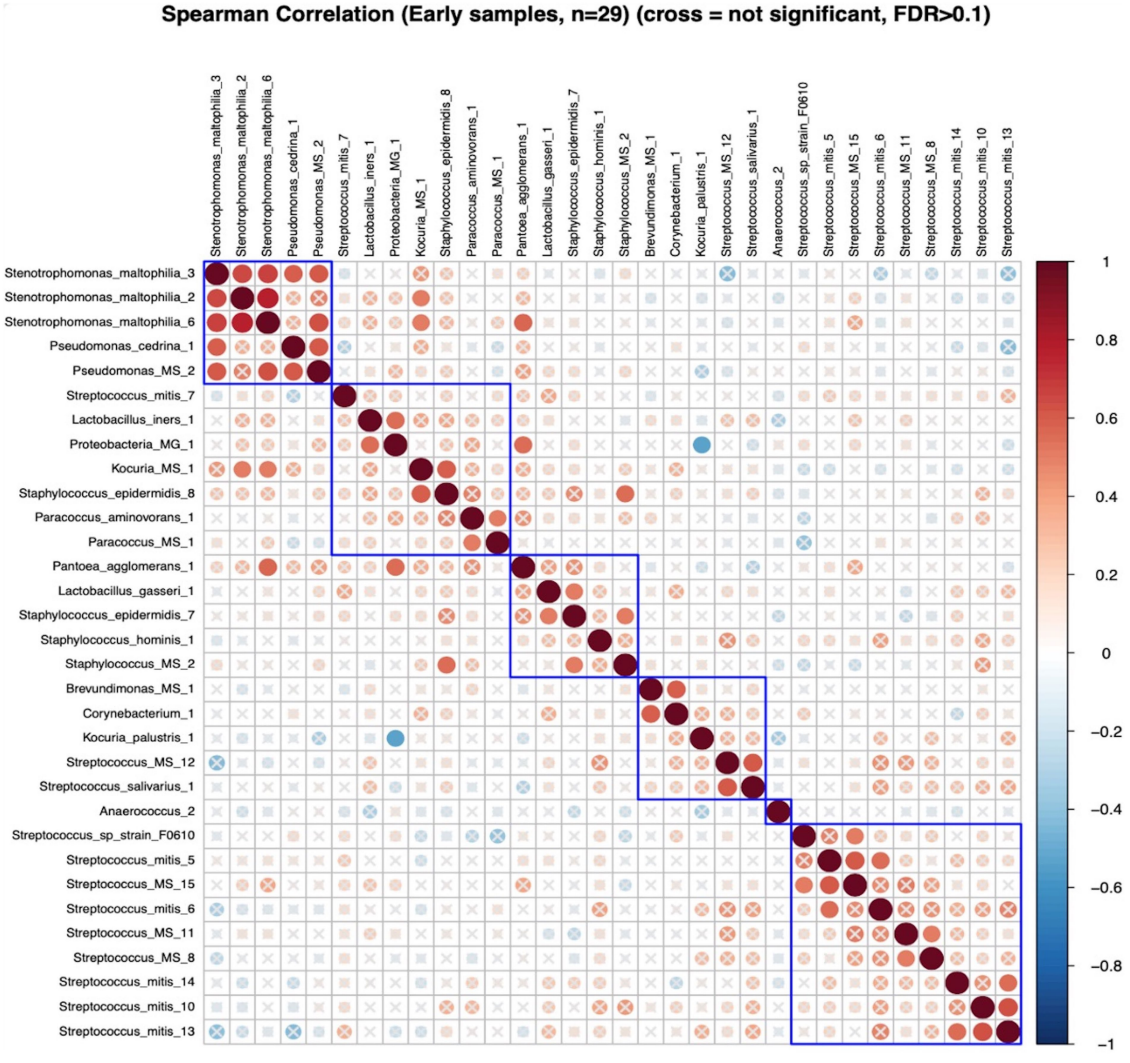
Hierarchical clustering of Spearman’s rank correlation heatmap between differentially abundant ESVs in early lactation. Red circles represent positive correlations and blue circles represent negative correlations. The blue boxes represent the clusters. Cluster 1: *Stenotrophomonas_maltophilia_3, Stenotrophomonas_maltophilia_2, Stenotrophomonas_maltophilia_6, Pseudomonas_cedrina_1,* and *Pseudomonas_MS_2*, Cluster 2: *Streptococcus_mitis_7, Lactobacillus_iners_1,* and *Proteobacteria_MG_1*, *Kocuria_MS_1, Staphylococcus_epidermidis_8, Paracoccus_aminovorans_1,* and *Paracoccus_MS_1*, Cluster 3:*Pantoea_agglomerans_1, Lactobacillus_gasseri_1, Staphylococcus_epidermidis_7, Staphylococcus_hominis_1,* and *Staphylococcus_MS_2*, Cluster 4: *Brevundimonas_MS_1, Corynebacterium_1, Kocuria_palustris_1, Streptococcus_MS_12,* and *Streptococcus_salivarius_1*, Cluster 5: *Streptococcus_sp_strain_F0610, Streptococcus_mitis_5, Streptococcus_MS_15, Streptococcus_mitis_6, Streptococcus_MS_11, Streptococcus_MS_8, Streptococcus_mitis_14, Streptococcus_mitis_10,* and *Streptococcus_mitis_13*.

**Figure 5:**
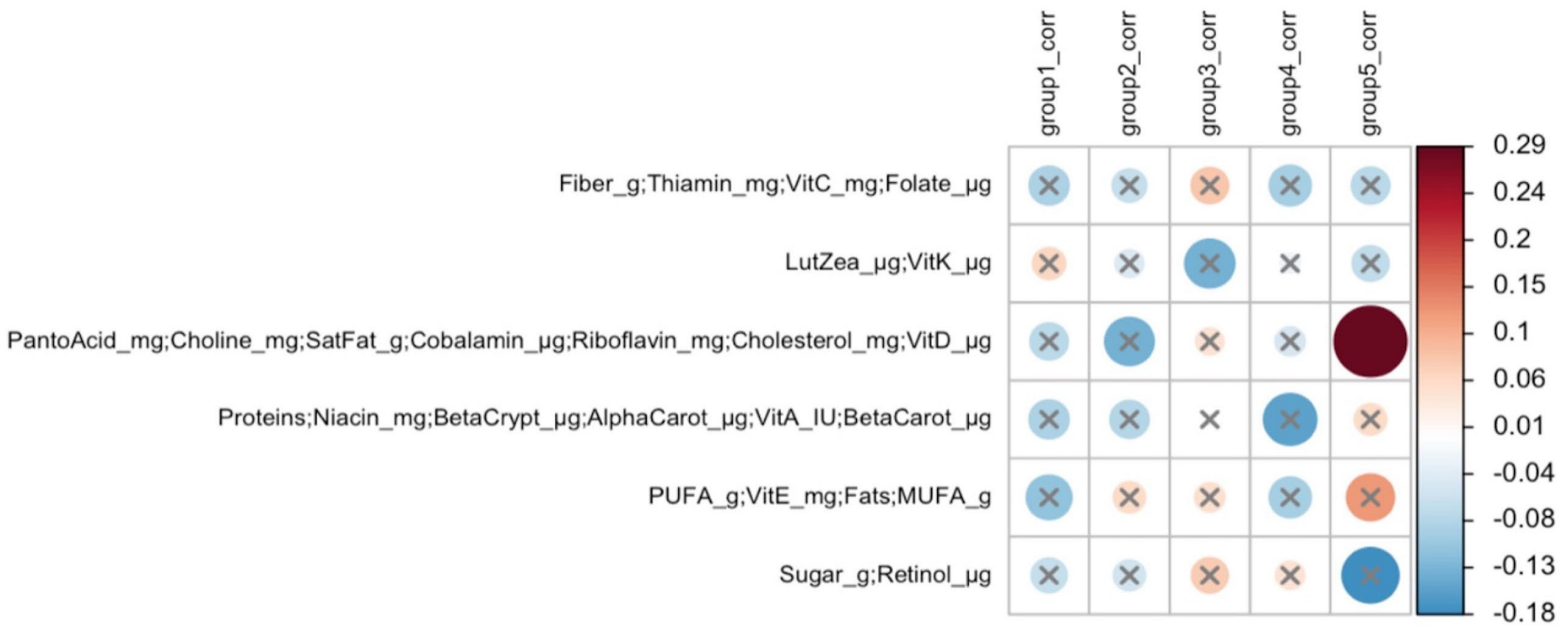
Heatmap of Mantel tests between maternal nutrient intake clusters (early lactation; Euclidean distance metric) and differentially abundant ESV clusters (based on Bray-Curtis distance metric). Red circles represent positive correlations and blue circles represent negative correlations. The intensity of the colors represents the degree of association. The solid circles represent significant correlations (FDR > 0.1). Cluster 5: *Brevundimonas_MS_1, Corynebacterium_1, Kocuria_palustris_1, Streptococcus_MS_12,* and *Streptococcus_salivarius_1* were significantly correlated with Cluster 3 of maternal nutrient intakes: pantothenic acid, choline, saturated fat, cobalamin, riboflavin, cholesterol, and vitamin D (FDR = 0.048; *r* = 0.3).

**Figure 6:**
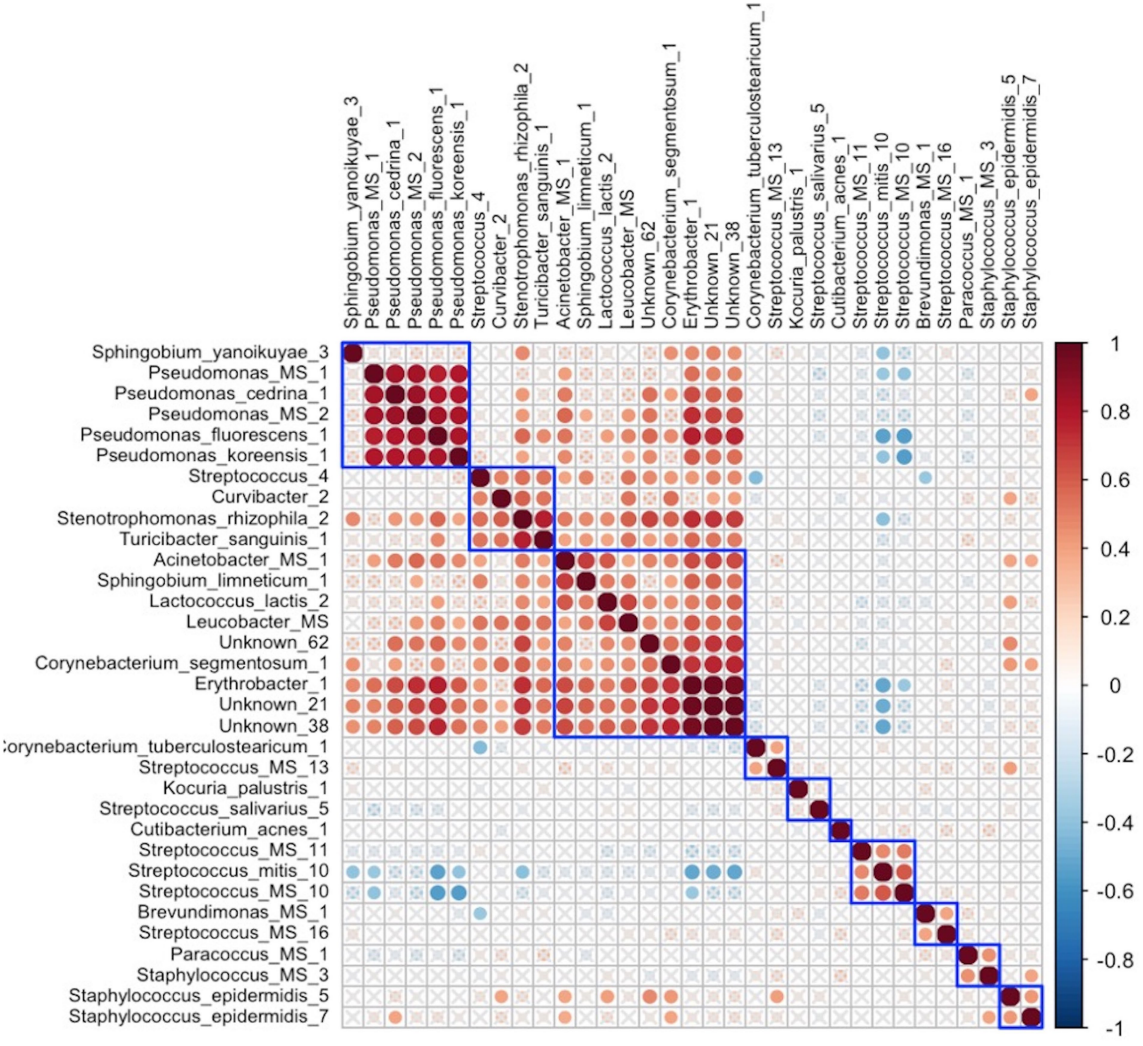
Hierarchical clustering of Spearman’s rank correlation heatmap between differentially abundant ESVs in late lactation. Red circles represent positive correlations and blue circles represent negative correlations. The blue boxes represent inferred clusters. Cluster 1: *Pseudomonas_MS_1, Pseudomonas_cedrina_1, Pseudomonas_MS_2, Pseudomonas_fluorescens_1,* and *Pseudomonas_koreensis_1*, Cluster 2: *Streptococcus_4, Curvibacter_2, Stenotrophomonas_rhizophila_2,* and *Turicibacter_sanguinis_1,* Cluster 3: *Acinetobacter_MS_1, Sphingobium_limneticum_1, Lactococcus_lactis_2, Leucobacter_MS, Unknown_62, Corynebacterium_segmentosum_1, Erythrobacter_1, Unknown_21,* and *Unknown_38,* Cluster 4: *Streptococcus_MS_11, Streptococcus_mitis_10,* and *Streptococcus_MS_10,* Cluster 5: *Brevundimonas_MS_1* and *Streptococcus_MS_16,* Cluster 6: included *Paracoccus_MS_1* and *Staphylococcus_MS_3*, and Cluster 7: *Staphylococcus_epidermidis_5* and *Staphylococcus_epidermidis_7*.

In early lactation, six differentially abundant species clusters were identified (Figure 4). One of the DA ESV clusters that included *Brevundimonas_MS_1, Corynebacterium_1, Kocuria_palustris_1, Streptococcus_MS_12, and Streptococcus_salivarius_1* was positively correlated with the maternal nutrient intake cluster that included pantothenic acid, choline, saturated fat, cobalamin, riboflavin, cholesterol, and vitamin D (FDR = 0.048; *r* = 0.3) (Figure 5). In late lactation, six differentially abundant species clusters were identified (Figure 6). However, none of the DA clusters were correlated with maternal nutrient intake clusters.

### Univariate analyses of DA HMM by infant growth and maternal nutrient intakes

**Figure 7:**
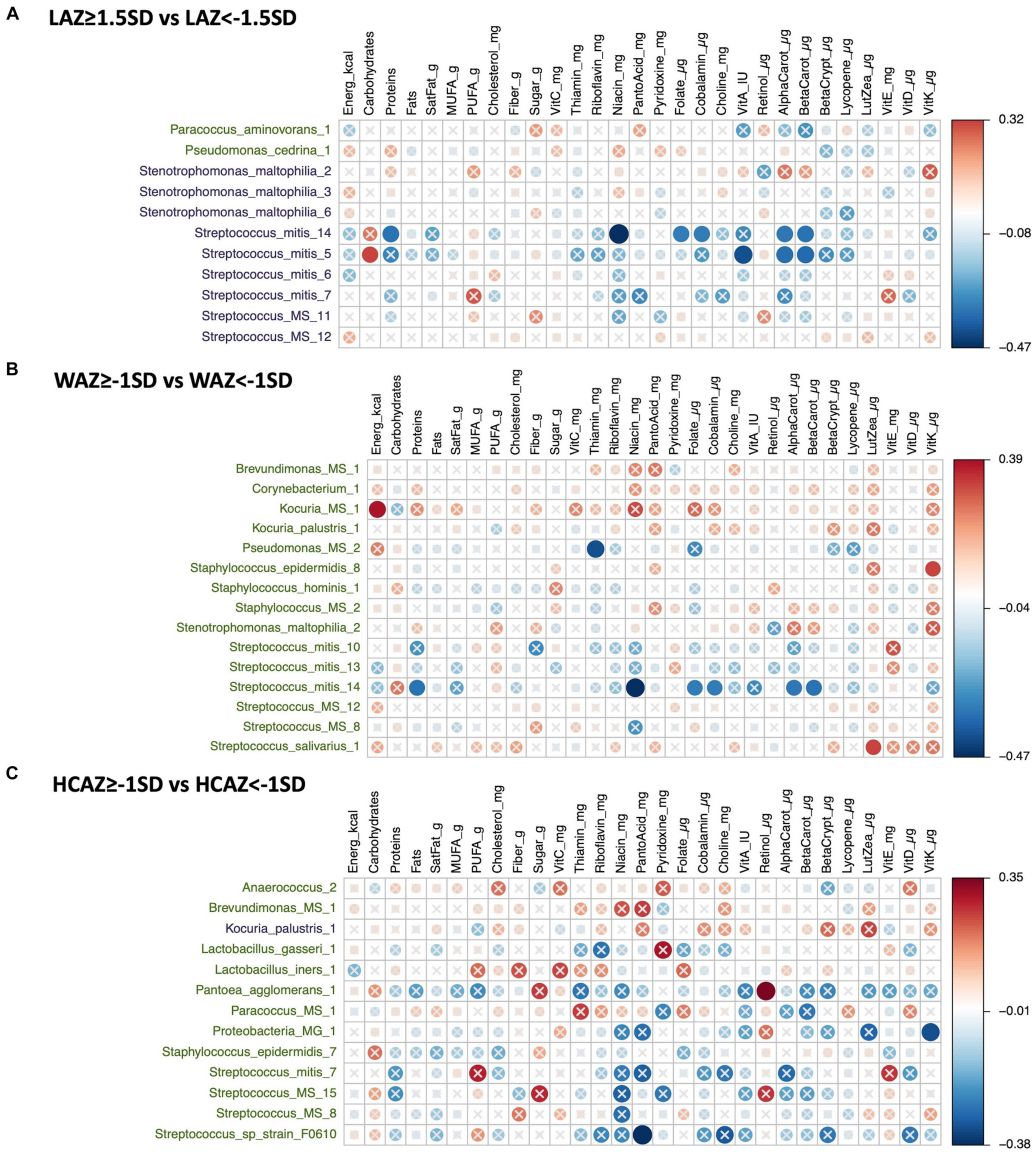
Heatmap of a univariate Spearman correlation matrix between the differentially abundant (DA) human milk microbiome (HMM) ESVs abundance (rlog; y-axis) *vs* maternal dietary information in early lactation (x-axis). **(A)** Shows the correlation between DA ESVs in the HMM of the mothers of non-stunted infant (LAZ ≥ −1.5SD) and the HMM of the mothers of mildly stunted infants (LAZ < −1.5SD) with maternal dietary information, **(B)** shows the correlation between DA ESVs in the HMM of the mothers of infants with normal weight (WAZ ≥ −1 SD) and the HMM of the mothers of infants with mild underweight (WAZ < −1SD) with maternal dietary information, and **(C)** shows the correlation between DA ESVs in the HMM of the mothers of infants with normal head-circumference (HCAZ ≥ −1 SD) and the HMM of the mothers of infants with mild smaller head-circumference (HCAZ < −1SD) with maternal dietary information. The black-colored taxa are the differentially abundant ESVs in the mild growth faltering groups: **(A)** LAZ < −1.5SD, **(B)** WAZ < −1SD, **(C)** HCAZ < −1SD. The green-colored taxa are the differentially abundant ESVs in the normal growth groups: **(A)** LAZ ≥ −1.5SD, **(B)** WAZ ≥ −1SD, **(C)** HCAZ ≥ −1SD. Red circles represent positive correlations and blue circles represent negative correlations. The intensity of the colors represents the degree of association between the HMM DA ESVs and nutrients as measured by Spearman’s correlations. The solid circles represent significant correlations (FDR > 0.1).

**Figure 8:**
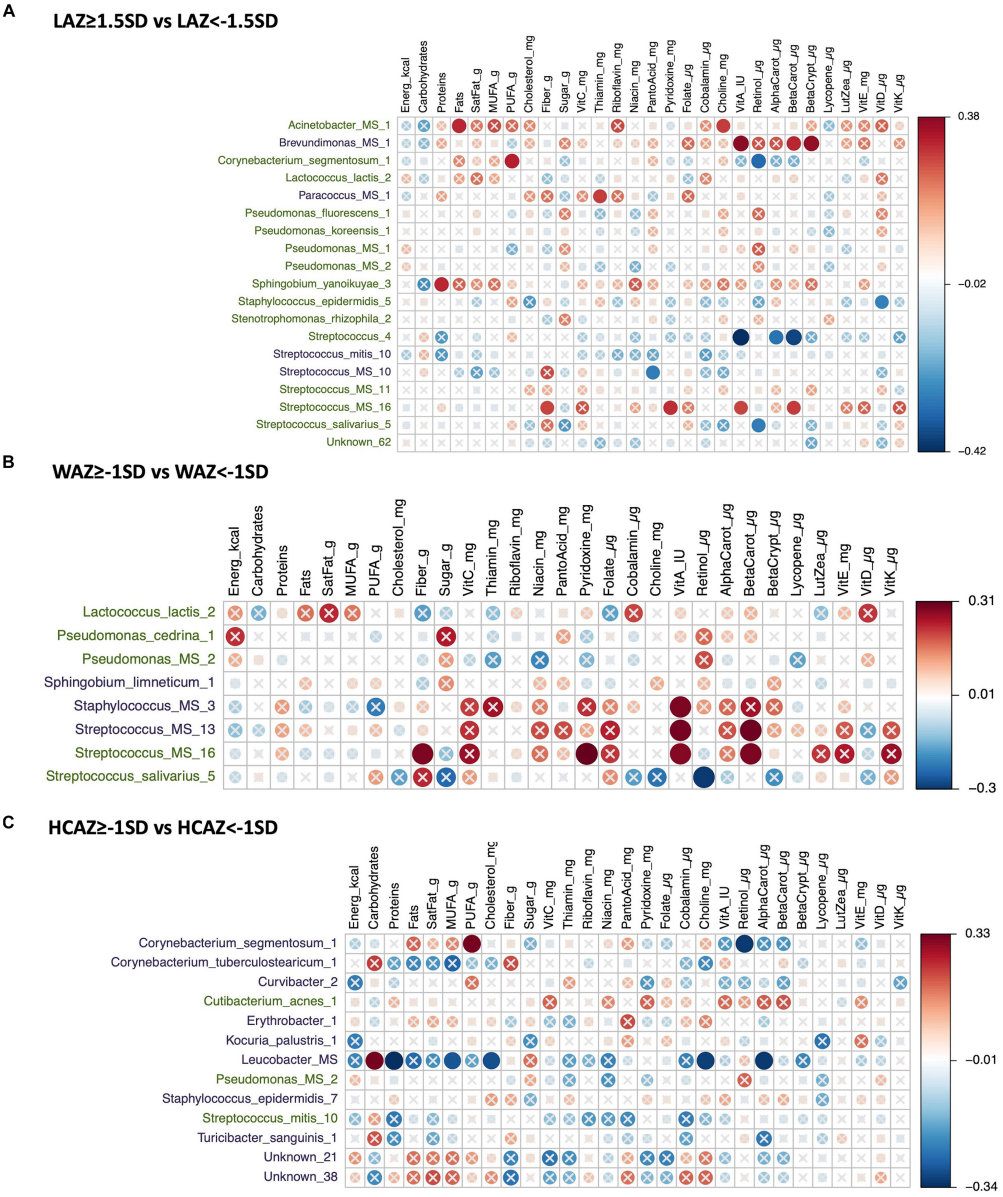
Heatmap of a univariate Spearman correlation matrix between the differentially abundant (DA) human milk microbiome (HMM) ESVs abundance (rlog; y-axis) *vs* maternal dietary information in late lactation (x-axis). **(A)** Shows the correlation between DA ESVs in the HMM of the mothers of non-stunted infant (LAZ ≥ −1.5SD) and the HMM of the mothers of midly-stunted infants (LAZ < −1.5SD) with maternal dietary information, **(B)** shows the correlation between DA ESVs in the HMM of the mothers of infants with normal weight (WAZ ≥ −1 SD) and the HMM of the mothers of infants with mild underweight (WAZ < −1SD) with maternal dietary information, and **(C)** shows the correlation between DA ESVs in the HMM of the mothers of infants with normal head-circumference (HCAZ ≥ −1 SD) and the HMM of the mothers of infants with mild smaller head-circumference (HCAZ < −1SD) with maternal dietary information. The black-colored taxa are the differentially abundant ESVs in the mild growth faltering groups: **(A)** LAZ < −1.5SD, **(B)** WAZ < −1SD, **(C)** HCAZ < −1SD. The green-colored taxa are the differentially abundant ESVs in the normal growth groups: **(A)** LAZ ≥ −1.5SD, **(B)** WAZ ≥ −1SD, **(C)** HCAZ≥ −1SD. Red circles represent positive correlations and blue circles represent negative correlations. The intensity of the colors represents the degree of association between the HMM DA ESVs and nutrients as measured by the Spearman’s correlations. The solid circles represent significant correlations (FDR > 0.1).

The DA species by infant growth groups [mildly stunted (LAZ < −1.5SD), non-stunted (LAZ ≥ −1.5SD), mild underweight (WAZ < −1SD), normal weight (WAZ ≥ −1SD), normal head-circumference (HCAZ ≥ −1SD), and smaller head-circumference (HCAZ < −1SD)] revealed multiple positive and negative correlations with maternal nutrient intakes in early lactation (Figures 7A–C) and late lactation (Figures 8A–C). Data are presented using heatmaps of Spearman rank-order correlation coefficient analysis (FDR < 0.1).

#### Correlations of DA ESVs and maternal nutrient intakes in early lactation with infant growth

##### LAZ

In total, 11 DA ESVs were identified; 9 were significantly more abundant in the mildly stunted (LAZ < −1.5SD) group compared with only 2 ESVs in the non-stunted (LAZ ≥ −1.5SD) group. Of the 9 DA species in the LAZ < −1.5SD, only 2, *Streptococcus_mitis, Streptococcus_mitis_14,* and *Streptococcus_mitis_5,* presented 10 distinct correlations with distinct maternal nutrient intakes. In terms of macronutrients, *Streptococcus mitis* was positively correlated with carbohydrates (FDR = 0.089; *r* = 0.32) but was negatively correlated with protein (FDR = 0.069; *r* = −0.34). In terms of micronutrients, both *Streptococcus_mitis_14* and *Streptococcus_mitis_5* were negatively correlated with alpha carotene (FDR = 0.073; *r* = −0.34) and (FDR = 0.058; *r* = −0.35) and beta carotene (FDR = 0.069; *r* = −0.34) and (FDR = 0.05; *r* = −0.37), respectively*. Streptococcus_mitis_14* was also negatively correlated with maternal intake of niacin (FDR = 0.009; *r* = −0.47), folate (FDR = 0.083; *r* = −0.33), and cobalamin (FDR = 0.087; *r* = −0.32), while *Streptococcus_mitis_5* was negatively correlated with maternal intake of vitamin A (FDR = 0.026; *r* = −0.41) (Figure 7A).

##### WAZ

Fifteen DA ESVs were identified as more abundant in the normal weight (WAZ ≥ −1SD) group; no DA species was identified in the mildly underweight (WAZ < −1SD) group. Of the 15 DA species, 4 DA species included two normal human microflora that were positively correlated with maternal nutrient intakes: *Streptococcus_salivarius_1* with lutein + zeaxanthin (FDR = 0.088; *r* = 0.32) and *Staphylococcus_epidermidis_8* with vitamin K (FDR = 0.094; *r* = 0.32). Two other correlated DAs were ambiguous species; these included *Kocuria_MS_1* which was positively correlated with energy intake (FDR = 0.038; *r* = 0.39) and *Pseudomonas_MS_2* which was negatively correlated with maternal intake of thiamin (FDR = 0.025; *r* = −0.41). The remaining DA species, *Streptococcus_mitis_14,* was also associated with the LAZ < −1.5SD group and presented similar correlations with maternal nutrient intakes that included negative nutrient correlations with both vitamin B and vitamin A precursors. These included negative correlations with niacin (FDR = 0.009; *r* = −0.47), folate (FDR = 0.083; *r* = −0.33), cobalamin (FDR = −0.087; *r* = −0.32), alpha carotene (FDR = 0.073; *r* = −0.34) and beta carotene (FDR = 0.069; *r* = −0.34), and protein (FDR = 0.069; *r* = −0.34) (Figure 7B).

##### HCAZ

In total, thirteen DA ESVs differed between the HCAZ groups; 12 were DA in the normal HC (HCAZ ≥ −1 SD) group compared with only 1 in the smaller HC (HCAZ < −1 SD) group. Three DA species of the normal HCAZ group were correlated with different maternal nutrient intakes. These correlations included two negative correlations: *Streptococcus_sp_strain_F0610* with pantothenic acid (FDR = 0.04; *r* = −0.38) and *Proteobacteria* at the genus-level and *Proteobacteria_MG_1* with vitamin K (FDR = 0.085; *r* = −0.32) and one positive correlation between *Pantoea_agglomerans_1* and retinol (FDR = 0.064; *r* = 0.35) (Figure 7C).

#### Correlations of DA ESVs and maternal nutrient intakes in late lactation with infant growth

##### LAZ

In total, 19 DA ESVs were identified between the LAZ groups; 15 were associated with the non-stunted (LAZ ≥ −1.5SD) group and 7 were correlated with maternal nutrient intakes. These DA species had both positive and negative correlations with diverse nutrients. Three were *Streptococcus* species that were correlated with maternal intakes of vitamin A and its precursors. These included *Streptococcus_4* that was negatively correlated with vitamin A (FDR = 0.011; *r* = −0.42), beta carotene (FDR = 0.021; *r* = −0.4), and alpha carotene (FDR = 0.067; *r* = −0.31), *Streptococcus_salivarius_5* was negatively correlated with retinol (FDR = 0.084; *r* = −0.3), and *Streptococcus_MS_16* was positively correlated with vitamin A (FDR = 0.097; *r* = 0.3), beta carotene (FDR = 0.091; *r* = 0.3), pyridoxine (FDR = 0.069; *r* = 0.31), and fiber (FDR = 0.093; *r* = 0.3). Other DA species were correlated with the fat component of the diet. These included *Acinetobacter_MS_1* that was positively correlated with fat (FDR = 0.065; *r* = 0.32) and choline (FDR = 0.088; *r* = 0.3), *Corynebacterium_segmentosum_1* was positively correlated with PUFA (FDR = 0.057; *r* = 0.32) and negatively correlated with retinol (FDR = 0.057; *r* = −0.32), and *Staphylococcus_epidermidis_5* was negatively correlated with vitamin D (FDR = 0.099; *r* = −0.3). Finally, one DA, *Sphingobium_yanoikuyae_3*, was positively correlated with protein (FDR = 0.066; *r* = 0.31) (Figure 8A).

Of the DA in the mildly stunted (LAZ < −1.5SD) group, we observed mainly correlations with maternal intakes of carotenes and vitamin B. These included positive correlations between *Brevundimonas_MS_1* and maternal intakes of beta-cryptoxanthin (FDR = 0.031; *r* = 0.4), beta carotene (FDR = 0.063; *r* = 0.32), and vitamin A (FDR = 0.025; *r* = 0.4) and between *Paracoccus_MS_1* and maternal intake of thiamin (FDR = 0.09; *r* = 0.3). Only *Streptococcus_MS_10* was negatively correlated with pantothenic acid (FDR = 0.08; *r* = −0.3) (Figure 8A).

##### WAZ

Eight DA ESVs were identified between the WAZ groups. Five were significantly more abundant in the normal weight group (WAZ ≥ −1SD) and three in the mildly underweight (WAZ < −1SD) group. Among these eight DA species, four species were correlated with maternal nutrient intakes, two DA species were associated with each group. The two DA species associated with the normal weight (WAZ ≥ −1SD) group were *Streptococcus* that were mainly correlated with maternal intake of vitamin A-related nutrients. *Streptococcus_MS_16* was positively correlated with beta carotene (FDR = 0.091; *r* = 0.3), vitamin A (FDR = 0.097; *r* = 0.3), pyridoxine (FDR = 0.069; *r* = 0.31), and fiber (FDR = 0.093; *r* = 0.3). In contrast, *Streptococcus_salivarius_5* was negatively correlated with retinol (FDR = 0.084; *r* = −0.3) (Figure 8B).

The two DA species associated with the mildly underweight (WAZ < −1SD) group were two that were positively correlated with vitamin A: *Staphylococcus_MS_3* (FDR = 0.092; *r* = 0.3) and *Streptococcus_MS_13* (FDR = 0.088; *r* = 0.3) and beta carotene (FDR = 0.08; *r* = 0.3) (Figure 8B).

##### HCAZ

Thirteen DA ESVs were identified between the HCAZ groups, 3 in the normal HC (HCAZ ≥ −1 SD) group and 10 in the smaller HC (HCAZ < −1 SD) group. All the identified correlations between maternal nutrient intakes and DA species were identified with only two DA species, *Corynebacterium_segmentosum_1* and *Leucobacter_MS,* which were uniquely associated with the smaller HC (HCAZ < −1 SD) group. These mainly negative correlations included *Corynebacterium_segmentosum_1* with retinol (FDR = 0.057; *r* = −0.32) and *Leucobacter_MS* with protein (FDR = 0.044; *r* = −0.34), choline (FDR = 0.059; *r* = −0.32), alpha carotene (FDR = 0.054; *r* = −0.33), MUFA (FDR = 0.088; *r* = −0.3), and cholesterol (FDR = 0.079; *r* = −0.3). Other positive correlations with these two DAs were *Corynebacterium_segmentosum_1* with PUFA (FDR = 0.057; *r* = 0.32) and *Leucobacter_MS* with carbohydrates (FDR = 0.054; *r* = 0.33) (Figure 8C).

## Discussion

A limited number of studies had previously reported associations between maternal nutrient intakes during lactation that had uncovered both multiple positive and negative associations with the HMM at the phylum and genus levels using correlation and cluster analyses (23–26). Since associations between the HMM and maternal nutrient intakes with infant growth parameters had not been explored, we also investigated associations of maternal nutrient intakes with previously established DA species that differed between infants with ‘normal infant z-scores’ defined as WAZ ≥ −1SD, LAZ ≥ 1.5SD, and HCAZ ≥ −1SD compared with infants experiencing growth faltering defined as mildly underweight (WAZ < −1SD), mildly stunted (LAZ < −1.5SD), and with smaller head circumferences (HCAZ < −1SD) by the stage of lactation (44, 45). Moreover, given that human milk continuously provides an infant with a dynamically changing community of commensal and potentially beneficial bacteria that differs between early and late lactation (43), we conducted our analyses at the species level during both early and late lactation.

Several novel findings emerged. First, in early lactation, cluster analyses revealed that infant z-scores were correlated with maternal energy intakes, and infant length parameters (height and LAZ) were correlated with maternal intakes of carbohydrates, riboflavin, and saturated fat. Second, with regard to maternal nutrient intake clusters, maternal intakes of lutein + zeaxanthin and vitamin K were correlated with infant growth parameters as a cluster that included infant weight and infant head circumference. However, in late lactation, infant anthropometric clusters were not correlated with maternal nutrient intakes or nutrient clusters. Third, univariate analyses in early and late lactation revealed multiple correlations between HMM DA taxa associated with infant growth and maternal nutrient intakes during lactation. Several of these were negative correlations of *Streptococcus* species with maternal intakes of vitamin B and vitamin A precursors during lactation. In late lactation, more environmental and ambiguous differentially abundant species were identified in the human milk microbial community compared with early lactation, and these taxa were correlated with several maternal nutrient intakes. Interestingly, the ambiguous environmental DA taxa uniquely identified in the smaller HC (HCAZ < −1 SD) group was *Leucobacter_MS*, which was negatively correlated with maternal nutrient intakes of choline that is required for the biosynthesis of the neurotransmitter acetylcholine.

### Maternal nutrient intakes and infant growth parameters

Maternal intakes between early and late lactation showed comparable dietary intakes that only differed in vitamin E, which was higher in early lactation; however, maternal dietary intakes of macronutrients differed between infant z-scores. In our study, maternal intake of dietary fat and/or fat components and vitamin E were associated with both infant WAZ and HCAZ but not LAZ during the first 6 months of lactation. Mothers of infants with normal weight (WAZ ≥ −1SD) had higher intakes of total calories and carbohydrates and higher intakes of energy and fat including total fat, MUFA, PUFA, and vitamin E. Mothers of infants with normal head-circumference (HCAZ ≥ −1 SD) also consumed higher MUFA and vitamin E. Previous studies investigating maternal intakes of dietary fat and fat components have described associations of higher dietary fat and fat component intakes with higher infant adiposity during the first 6 months of lactation (70) and higher lipid components in breast milk with infant of length and weight up to 12 months postpartum (71).

There is also evidence that maternal concentrations of tocopherols also have been positively associated with better infant growth for at least one parameter (weight, length, head circumference (72), and percentile rankings for weight, length, and head circumference at birth (73)). There is also evidence in an older investigation that plasma concentrations of oxidative metabolites were higher, and levels of vitamins A, C, and E were lower in low-birth-weight infants compared with a normal birth-weight control group (74). More recent evidence continues to support the concept that oxidative stress during pregnancy may be associated with low birth weight, preterm delivery, and oxidative stress-related diseases (75). In our study, maternal dietary intakes of vitamin E were higher among mothers of infants with normal WAZ and HCAZ compared with underweight infants and infants with smaller HCAZ. Collectively, our findings highlight that the importance of adequate maternal energy, dietary fat, and vitamin E intakes in lactating mothers from marginalized communities that, if not adequate, do have consequences for early infant growth and can be associated with growth faltering and lower WAZ and HCAZ during the first 6 months of lactation.

### Cluster analyses associating nutrients with anthropometry

In early lactation, cluster analyses revealed that the infant z-scores were correlated with maternal energy intakes, and infant length parameters (height and LAZ) were correlated with maternal intakes of carbohydrates, riboflavin, and saturated fat. Among the Guatemalan population, higher energy intake is derived mainly from carbohydrates with lower intakes of animal proteins and dietary fat (76, 77). This was reflected in our study by the high carbohydrate intake (78% of the total energy) and the low intakes of saturated fat (2.8% of the total energy intake). When compared with the Acceptable Macronutrient Distribution Range (AMDR) for macronutrients, carbohydrate intakes in our population were above the upper AMDR limit of 65% and below the upper limit for saturated fat of <10%. Moreover, higher intakes of carbohydrates in our study population were negatively correlated not only with protein and fat intakes as a percentage of overall energy intakes but also with individual dietary lipids including saturated fat, MUFA, cholesterol, and micronutrients including two fat-soluble vitamins E and D and a range of vitamin B and vitamin C that slightly differed between early and late lactation.

Maternal nutrient intakes of lutein + zeaxanthin and vitamin K emerged as a cluster that was correlated with the infant growth cluster of infant head circumference, infant weight, and infant age in days. Lutein and zeaxanthin are fat-soluble dietary carotenoids found in dark green leafy vegetables and egg yolks and corn (78), which are principle components of the Mayan diet (76, 77). They have powerful anti-inflammatory properties that reduce oxidative stress and increase antioxidant capacities in newborns when supplemented during the first days of life (79, 80). Lutein and zeaxanthin are found with high concentrations in the brain tissues, and they were associated with cognitive function in young and older adults (81–84). In preterm infants, lutein was the prevalent carotenoid in the developing brain, and its concentration was lower in preterm neonates compared with term neonates (85), and when supplemented in healthy infants, it supported physical growth including HC growth (86). Our findings were associated with improved HC growth among infants of mothers with higher lutein and zeaxanthin intakes in early lactation.

### Associations of maternal dietary intakes with the HM and the HMM

Studies exploring associations of maternal nutritional status with the vitamin composition of breastmilk have revealed that some vitamins might be inadequate for the growing infant, as a consequence of compromised maternal nutritional status (87–97) and poor maternal diet (97–100). However, the latest systematic review on HM components and infant anthropometry in the first 2 years of life among term infants uncovered no eligible studies for several vitamins including, riboflavin, C, D, E, and K and concluded that for others, the available evidence was largely inconclusive and further studies were needed (101). In our exploratory study in Guatemala where most women breastfeed but infants experience growth faltering by 3 months (40, 102), we were able to observe correlations between selected nutrient intakes, the HMM, and infant growth.

Researchers had previously associated several macronutrients including protein, fat, and fiber with HMM at the phylum and genera levels. Reportedly, maternal protein intakes have been associated with higher HM *Gemella* (25, 26), and maternal fiber intakes have been associated with one member of the Bacillota phylum and negatively with *Bifidobacterium*, *Ctibacterium, and Serratia.* At the genus level, total dietary fiber and insoluble dietary fiber intakes were negatively associated with *Finegoldia* and *Streptococcus* (23, 24), whereas *Veillonella* was positively associated with total fiber and soluble and insoluble fibers (26). Furthermore, these studies reported several associations between maternal intakes of the different dietary fat components (23–26) and vitamin B (23, 25) with the HMM. For example, maternal intakes of MUFA were negatively correlated with *Corynebacterium* (25) and *Sediminibacterium* at the genus level (26). In addition, maternal intakes of PUFA were positively correlated with *Gemella* (25), *Bifidobacterium,* and *Atopobium* (23) and negatively correlated with *Acinetobacter* (24), *Bifidobacterium, Serratia*, and *Ralstonia* (26). Furthermore, several B vitamins were reported to have negative correlations with HMM including, pantothenic acid with *Streptococcus* (25) and thiamin and folate with *Granulicatella* (23). In our study, we took the analysis a step further, and we uncovered maternal dietary intake associations at the species level.

### Maternal nutrient intakes and DA human milk microbiome by infant growth

Our exploratory study uncovered previously unidentified correlations among maternal macronutrient and micronutrient intakes, infant growth in breast-fed infants, and the HMM in both early and late lactation. In both stages, univariate analyses of the DA HMM species by infant growth z-scores revealed multiple correlations with specific maternal nutrient intakes.

In our population, in early lactation, *Streptococcus_mitis* dominated the DA species in the mildly stunted (LAZ < −1.5SD) group with four distinct *Streptococcus_mitis species (Streptococcus_mitis_5, Streptococcus_mitis_6, Streptococcus_mitis_7, and Streptococcus_mitis_14)* and was positively correlated with maternal carbohydrate intakes, which are associated with plaque oral microbiome abundance and diversity (103–105). *Streptococcus_mitis* is identified as an oropharynx bacterium that has been found in the infant oral microbiome within a few days after birth (106). However, it is responsible for the development of dental caries (107, 108), and it is considered an opportunistic pathogen (109, 110), and it has demonstrated the highest level of penicillin resistance (111). Furthermore, there is existing evidence to support this negative association between oral bacteria involved in dental plaque with poor prenatal outcomes for infant birth-weight (112, 113). Our previous findings showed that the majority of women had periodontal disease in the forms of caries (78%) and/or gingivitis (66%) (46), suggesting potential impacts of the oral potentially pathogenic bacteria in the HMM on infant growth during lactation that might be exacerbated by a low-quality diet and higher carbohydrate intakes.

In addition, in early lactation, other DA *Streptococcus* species were negatively correlated with maternal intakes of B vitamins and vitamin A precursors regardless of the growth classification. *Streptococcus_sp_strain_F0610* in the normal head circumference (HCAZ ≥−1SD) group was negatively correlated with maternal intake of pantothenic acid. Although this finding might be contradictory to what is known about *Streptococci* that require vitamin B5 for their growth *in vitro* (114), this finding is consistent with the study by Williams et al. (25) who reported that maternal pantothenic acid intake during lactation was negatively related to *Streptococcus* at the genus level (25). Pantothenate is involved in the Coenzyme A (CoA) synthesis, which functions as an acyl carrier and is a required cofactor for all living cells. Pantothenate analogs also have been shown to markedly suppress the growth of some opportunistic pathogens by inhibiting phosphorylation activity, which catalyzes the first step of the CoA biosynthetic pathway in specific bacteria, such as *Staphylococcus* species that inhabit human skin including *Staphylococcus epidermidis, Staphylococcus saprophyticus, and Staphylococcus aureus* (115, 116), the most common microorganism identified in mastitis among lactating mothers (117, 118), suggesting pantothenate to be effective in preventing infections by opportunistic pathogens. In our study, *Staphylococcus aureus* was not identified as DA by infant growth and was not correlated with maternal intake of vitamin B5, possibly due to our exclusion criteria of mastitis and sub-clinical mastitis which might have yielded low abundance of *Staphylococcus aureus* in our study population.

In late lactation, most *Streptococcus species* exhibited similar patterns to those observed in early lactation; however, some exceptions were observed among the *Streptococcus ambiguous* species. In late lactation, a *Streptococcus species, Streptococcus_MS_10,* in the mildly stunted (LAZ < −1.5SD) group was negatively correlated with pantothenic acid, and *Streptococcus_salivarius_5* in the HMM of mothers of infants with WAZ ≥ −1SD was negatively correlated with maternal intake of retinol. However, there were two exceptions among the species of *Streptococcus ambiguous* that were positively correlated with maternal nutrient intake. These included the species *Streptococcus_MS_16,* which was DA in both the non-stunted (LAZ ≥ −1.5SD) group and the normal weight (WAZ ≥ −1SD) group, and was positively correlated with maternal intake of pyridoxine, beta carotene, fiber, and vitamin A, and the DA *Streptococcus_MS_13* in the latter group was positively correlated with maternal intakes of vitamin A and beta carotene.

In late lactation, one interesting correlation was observed with a non-Streptococcus ambiguous species. Interestingly, *Leucobacter_MS*, which were uniquely associated with a smaller head circumference (HCAZ < −1SD), was negatively correlated with maternal choline intakes, a nutrient required for the biosynthesis of the neurotransmitter acetylcholine. *Leucobacter_MS* was also negatively correlated with maternal intakes of protein, alpha-carotene, MUFA, and cholesterol but was positively correlated with carbohydrate intakes, possibly suggesting a higher abundance with a low-quality maternal diet.

### Environmental bacteria in human milk linked to maternal intake and infant growth

In late lactation, HMM was more diverse and included ambiguous and environmental species. The latest systematic review of the HMM species origin reported that more than half of the studied species were first isolated from the environmental sources, suggesting their normal presence in human milk (119). In rural agricultural communities, which was the case for our study population, environmental bacteria contribute to the gut microbiome (120), which is one of the main sources of the HMM (9–13). In our study in late lactation, environmental bacteria were found in the HMM of both LAZ and WAZ infant subgroups (normal LAZ, mildly-stunted, normal WAZ, and mild underweight). Guatemalan mothers participate in fruit and vegetable harvesting (46), and they have a high interaction with the Guatemalan-rich soil (121), which would support the presence of environmental bacteria in the Guatemalan mother’s milk and might be considered as integral components of breast milk in agricultural societies as previously reported (119).

In our population, we observed both non-pathogenic and potentially pathogenic environmental bacteria among infant z-score groups, which were also correlated with maternal nutrient intakes. In late lactation, the ambiguous species, *Leucobacter_MS,* was uniquely differentially abundant in the smaller HC (HCAZ < −1 SD) group. This ambiguous species could be either *Leucobacter_komagatae* or *Leucobacter_aridicollis,* and both have been isolated from contaminated plant and water environments (122, 123). Interestingly, this taxon was negatively correlated with the maternal intake of choline, which is a nutrient required to produce the neurotransmitter acetylcholine, and alpha-carotene, and its higher plasma levels were associated with higher cognitive scores in adults (124). *Leucobacter_MS* was also negatively correlated with protein, alpha-carotene, MUFA, and cholesterol, possibly suggesting a higher abundance with a low-quality maternal diet. This negative correlation is consistent with the finding of the lower intakes of MUFA among mothers of infants with smaller head circumferences (HCAZ < −1 SD). On the other hand, *Pantoea_agglomerans_1* was DA in the normal head circumference (HCAZ ≥ −1 SD) group and was positively correlated with maternal retinol intake. *Pantoea_agglomerans* is a plant non-pathogenic bacterium to humans that rarely causes opportunistic human infections (125–127).

Among the DA species between the LAZ and WAZ comparative groups, several ambiguous and environmental taxa were correlated with maternal nutrient intakes. Two ambiguous taxa in the non-stunted (LAZ ≥ −1.5SD) group were positively correlated with maternal nutrient intakes. The first one was *Acinetobacter_MS_1*, which could be either the commensal human skin and mucosal colonizer *Acinetobacter_lwoffii* (formerly known as *Acinetobacter calcoaceticus* var. *lwoffii*) (128, 129) or the non-pathogenic environmental species *Acinetobacter_guillouiae*, which was isolated from soil and water (Yoon et al., 2014). *Acinetobacter_MS_1* was positively correlated with higher maternal intakes of fat and choline. In contrast, among the DA in the mildly stunted (LAZ < −1.5SD) group, two ambiguous environmental taxa that were associated with soil and water were positively correlated with maternal dietary intake. *Brevundimonas_MS_1* was positively correlated with maternal intakes of vitamin A, beta-cryptoxanthin, and beta-carotene. This ambiguous taxon could be either *Brevundimonas_vesicularis or Brevundimonas_nasdae* that were isolated from the soil and aquatic environments, respectively (130–132). In addition, *Paracoccus_MS_1* was positively correlated with maternal intake of thiamin. This ambiguous taxon could be either *Paracoccus_carotinifaciens or Paracoccus_marcusii.* The latter has been shown to improve growth, elevate antioxidant properties, suppress the expression of some inflammatory genes in marine animals (133, 134), and increase the probiotic properties of whey proteins (135).

## Strengths and limitations

Our study included major strengths. First, although our study had a cross-sectional design, milk samples and metadata were collected at two stages of lactation, allowing us to establish the association between the milk microbiome and maternal nutrient intakes. Second, the homogeneity of our cohort might have been an asset, as our population included healthy mothers, free from sub-clinical mastitis, did not take antibiotics, and complied with the WHO recommendations to breastfeed for 6 months. These factors are known to affect the milk microbiome ecosystem but were absent in our study. Third, mothers were recruited from eight distinct remote communities to minimize the possibility of exchanging microbes among mothers. Fourth, milk samples yielded sufficient DNA extraction capturing six million sequence reads, which allowed proceeding with this secondary analysis. Fifth, we used the ANCHOR method (62), which uses multiple samples and multiple reference databases with the criteria of >99% for identity and coverage to annotate bacteria. These very high criteria provide high confidence and resolution for the annotation at the species level, which maximizes biological discovery. Sixth, we used the hypervariable regions V1–V3 for the sequencing. These regions have been shown to be highly informative and have produced comparable results to the full-length 16S rRNA V1–V9 in the human gut microbiome samples at species level when used in conjunction with an appropriate identity thresholds (136). In our study, we combined the ANCHOR pipeline, which provided high resolution at the species level microbial identification in conjunction with >99% identity and coverage threshold. However, we understand that species should be considered putative even when single species sequences share 100% 16S rRNA gene fragment similarity because many species remain poorly characterized and mistakes exist in major repositories. Seventh, for the first time, this study bridged maternal diet during lactation and the human milk microbiome with infant growth during the first 6 months of life, which could be mediated by the influence of the human milk microbiome on the infant gut microbiome.

We also recognize that our study had several limitations. First, our study was cross-sectional in design, thereby limiting us to correlation analyses and therefore we cannot infer causality. Second, this study might be under-powered, as originally, as it had been powered to detect only differences in infant growth in early and late lactation and not to detect associations with maternal nutrient intakes. Third, some of the nutrients might require more 24-h recalls to estimate the usual intake. Fourth, maternal diet may influence milk composition, providing a pathway through which maternal diet may directly influence offspring growth (137). However, limited evidence is available on the relationship between maternal diet during lactation on infant postnatal growth or adiposity. In our study, we did not assess the milk nutrient compositions. Fifth, in this study, we used the 27F/533R primer. This primer can amplify the core human milk genus *Cutibacterium* (60, 61, 138); however, it has limitations for amplifying *Bifidobacterium* genus (139). Sixth, due to the lack of studies that associate the maternal diet with human milk microbiome and infant growth, the exploratory nature of this study, and the potential significant biological role of some species, correlations with FDR < 0.1 and (rs) ≤0.3 or (rs) ≥ −0.3 were included in our study. We used FDR instead of value of *p* to limit false positive correlations in our study; however, our study might have included some.

Finally, our current study also addressed some concerns found in earlier studies including application of aseptic techniques such as not using breast pumps during milk sample collection and adding cross-contamination control measures performed during recruitment and at different stages of laboratory and bioinformatics analyses. We also collected the 24-h dietary recall data and the milk samples concurrently and controlled our inclusion criteria by excluding antibiotic use or mothers with diabetes. Finally, the milk samples in our study were collected within a time period to ensure that we did not collect colostrum, which might have different microbial characteristics and mothers who did not have elevated sodium:potassium ratios, indicative of breast inflammation and sub-clinical mastitis.

## Conclusion

To the best of our knowledge, this study is the first to explore associations between maternal diet, the HMM, and infant z-scores and highlights an overlooked contribution of maternal diet and the HMM on early infant growth that can be mediated by the HMM during early and late lactation. In this exploratory study, we observed that maternal nutrient intakes during lactation were correlated with both infant growth parameters and milk microbiome. However, the presence of multiple positive and negative correlations suggests complex interactions between maternal nutrient intakes, the milk microbiota, and infant growth. Further research is required to understand how human milk micronutrients and microbiomes work independently and together to influence infant growth and identify new avenues for future maternal, newborn, and infant microbiome and nutritional interventions.

## Data availability statement

The raw sequence data has been deposited at the European Genome-Phenome Archive (EGAD00001004160) and are available upon request to KK, kristine.koski@mcgill.ca.

## Ethics statement

The study began as a collaboration between McGill University and the Center for Studies of Sensory Impairment, Aging, and Metabolism (CeSSIAM), a research organization based in Guatemala. Ethical approvals were obtained from ethics boards at McGill University and at CeSSIAM. Further approvals were obtained from community leaders and the local authorities at the Ministry of Health in Guatemala. The studies were conducted in accordance with the local legislation and institutional requirements. Written informed consent for participation in this study was provided by the participants’ legal guardians/next of kin. Written informed consent was obtained from the individual(s), and minor(s)’ legal guardian/next of kin, for the publication of any potentially identifiable images or data included in this article.

## Author contributions

TA: Conceptualization, Data curation, Formal analysis, Methodology, Writing – original draft, Writing – review & editing. EG: Data curation, Formal analysis, Methodology, Software, Visualization, Writing – review & editing. NS: Funding acquisition, Methodology, Writing – review & editing. MV: Methodology, Writing – review & editing. KK: provided funding for the 16s rRNA analysis, Writing – original draft, Writing – review & editing.
