# Supplementary figures and images for: Human milk microbiome: associations with maternal diet and infant growth
Source: Front Nutr. 2024 Mar 11;11:1341777. doi: 10.3389/fnut.2024.1341777 (PMC10962684; doi:10.3389/fnut.2024.1341777)

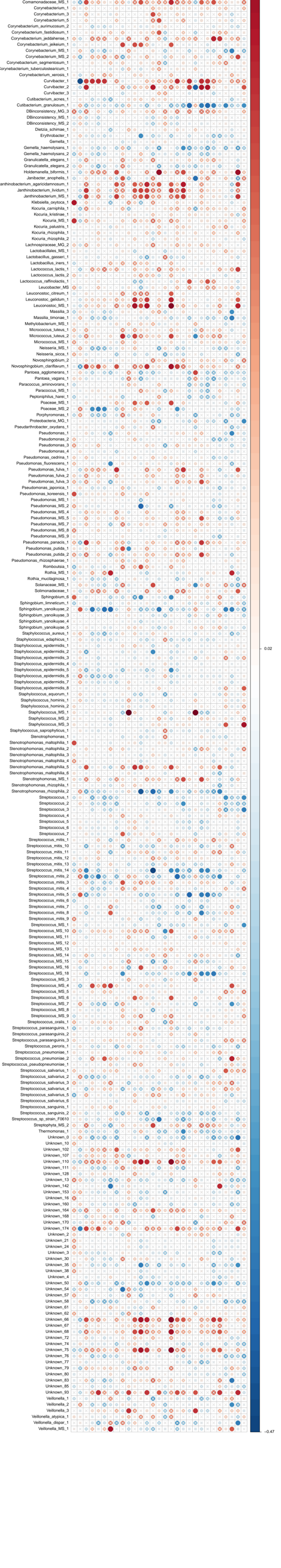

Supplement: Supplementary file 2 [file Data_Sheet_2.pdf]

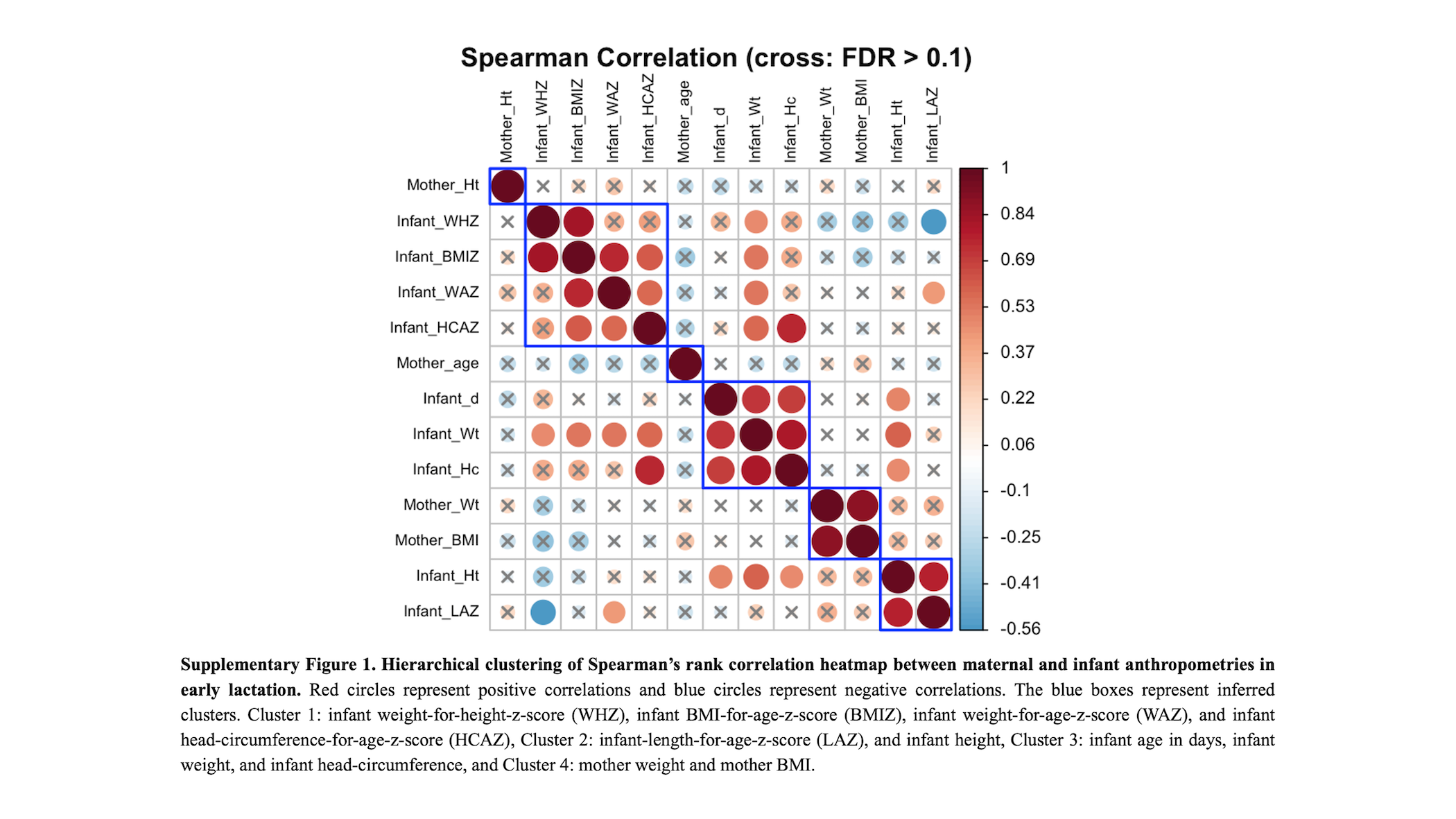

Supplement: Supplementary file 4 [file Image_1.tiff]

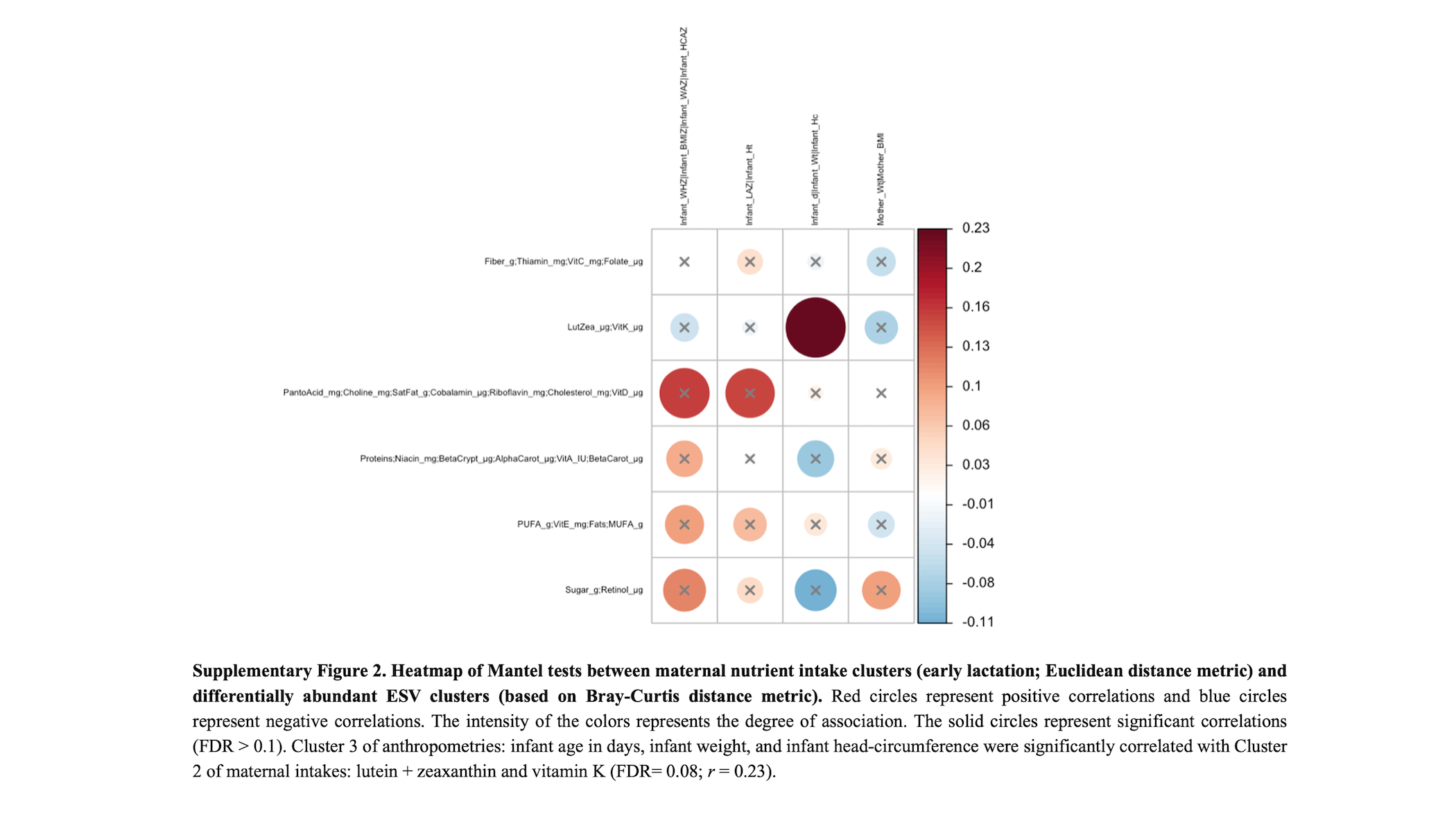

Supplement: Supplementary file 5 [file Image_2.tiff]
