# Supplementary material for: Human milk microbiome: associations with maternal diet and infant growth
Source: Front Nutr. 2024 Mar 11;11:1341777. doi: 10.3389/fnut.2024.1341777 (PMC10962684; doi:10.3389/fnut.2024.1341777)

Supplementary File 3. Heatmap of a univariate Spearman correlation matrix between the human milk microbiome (HMM) ESVs abu[illegible] vs maternal dietary information in late lactation (x-axis). Heatmap showing Spearman's correlations between human milk microbiom[illegible] and maternal nutrient intakes. Rows and columns are clustered by Euclidean distance and Ward linkage hierarchical clustering. Re[illegible] positive correlations and blue circles represent negative correlations. The intensity of the colors represents the degree of associatio[illegible] between the HMM taxa and the nutrients. The solid circles represent significant correlations (FDR > 0.1).

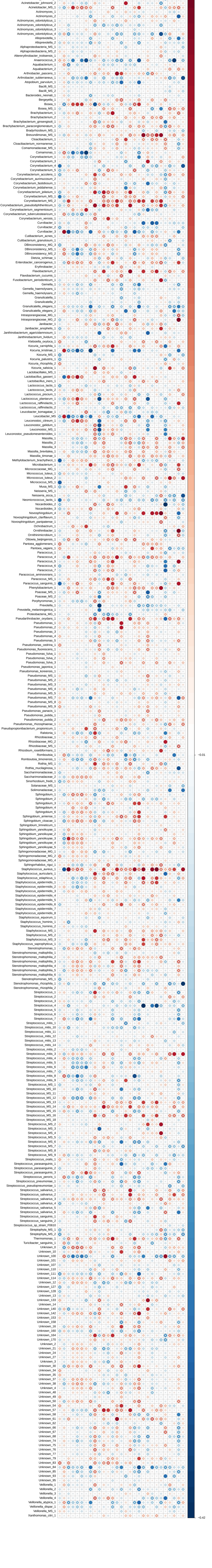

Supplement: Supplementary file 3 [file Data_Sheet_3.pdf]
